# Autocrine regulation of tumor cell repopulation by Hsp70-HMGB1 alarmin complex

**DOI:** 10.1186/s13046-023-02857-0

**Published:** 2023-10-25

**Authors:** Dmitry V. Sverchinsky, Bashar A. Alhasan, Marina A. Mikeladze, Vladimir F. Lazarev, Liubov S. Kuznetcova, Alisa V. Morshneva, Alina D. Nikotina, Amr Ziewanah, Lidia V. Koludarova, Tatiana Y. Starkova, Boris A. Margulis, Irina V. Guzhova

**Affiliations:** 1 Department of Molecular and Cellular Interaction, Institute of Cytology of Russian Academy of Sciences, Tikhoretsky prospect, 4, St. Petersburg, 194064 Russia; 2 Present Address: University of Kaiserslautern, Erwin-Schrödinger-Straße 52, 67663 Kaiserslautern, Germany; 3 Present Address: Institute of Biotechnology, University of Helsinki, Viikinkaari 5, Biocenter 2, Helsinki, 00790 Finland

**Keywords:** Tumor relapse, Alarmins, Hsp70, HMGB1, Protein complex

## Abstract

**Background:**

Cancer recurrence is regulated by a variety of factors, among which is the material of dying tumor cells; it is suggested that remaining after anti-cancer therapy tumor cells receive a signal from proteins called damage-associated molecular patterns (DAMPs), one of which is heat shock protein 70 (Hsp70).

**Methods:**

Two models of tumor repopulation were employed, based on minimal population of cancer cells and application of conditioned medium (CM). To deplete the CMs of Hsp70 affinity chromatography on ATP-agarose and immunoprecipitation were used. Cell proliferation and the dynamics of cell growth were measured using MTT assay and xCELLigence technology; cell growth markers were estimated using qPCR and with the aid of ELISA for prostaglandin E detection. Immunoprecipitation followed by mass-spectrometry was employed to identify Hsp70-binding proteins and protein-protein interaction assays were developed to reveal the above protein complexes.

**Results:**

It was found that CM of dying tumor cells contains tumor regrowth-initiating factors and the removal of one of them, Hsp70, caused a reduction in the relapse-activating capacity. The pull out of Hsp70 alone using ATP-agarose had no effect on repopulation, while the immunodepletion of Hsp70 dramatically reduced its repopulation activity. Using proteomic and immunochemical approaches, we showed that Hsp70 in conditioned medium binds and binds another abundant alarmin, the High Mobility Group B1 (HMGB1) protein; the complex is formed in tumor cells treated with anti-cancer drugs, persists in the cytosol and is further released from dying tumor cells. Recurrence-activating power of Hsp70-HMGB1 complex was proved by the enhanced expression of proliferation markers, Ki67, Aurka and MCM-10 as well as by increase of prostaglandin E production and autophagy activation. Accordingly, dissociating the complex with Hsp70 chaperone inhibitors significantly inhibited the pro-growth effects of the above complex, in both in vitro and in vivo tumor relapse models.

**Conclusions:**

These data led us to suggest that the abundance of the Hsp70-HMGB1 complex in the extracellular matrix may serve as a novel marker of relapse state in cancer patients, while specific targeting of the complex may be promising in the treatment of cancers with a high risk of recurrence.

**Supplementary Information:**

The online version contains supplementary material available at 10.1186/s13046-023-02857-0.

## Background

The phenomenon of cancer relapse is typical of a great number of tumor cells, with enhanced resistance generated due to intrinsic mutations, stressful conditions in a host organism, the need to evade an immune response, or the action of anti-cancer therapy. In many cases, tiny populations of tumor cells constituting so called minimal residual disease, acquire a dormant phenotype that is characterized by extremely low growth and metabolic activity and an increased level of autophagy [1]. Such cells, known as disseminated tumor cells or drug-tolerant persisters, demonstrate the attributes of cancer stem cells (CSCs) [2, 3]. Irrespective of their origin, dormant cells persist in special niches as single cells or sparse colonies (micrometastases) for an indefinite period of time. After receiving a signal, most often from the tumor microenvironment (TME), quiescent cells trigger their intrinsic mechanisms to induce proliferation and terminate the relapse cycle [4]. The remnants of dying tumor cells or so-called DAMPs (Damage-Associated Molecular Patterns) are found among the inducers of repopulation; these proteins, sometimes also called alarmins, were found to be released from dead or living cells [5]. However, it is not entirely clear how the debris of dying tumor cells or alarmins can induce proliferation and further relapse. Recent reports indicate that DAMPs may affect the activity of TME immune cells (macrophages) or adjacent cancer cells. Some of the most important alarmins are HMGB1 (High Mobility Group Box 1), Hsp70 (Heat shock protein 70 kD) and calreticulin [6]. It has been established that HMGB1, after anti-tumor therapy, changes its conformation and translocates from the nucleus to the cytoplasm and then into the extracellular matrix [7]. The protein was found to affect naive cancer cells using RAGE or TLR2/4 receptors, which induce the production of prostaglandin E (PGE) through the activation of NF-kappaB, which induces a new wave of proliferation. In fact, the chain of events mediated by HMGB1 is more complicated and involves other regulatory pathways associated with caspase 3 activation, for instance and the induction of pro-inflammatory cytokine production by TME-resident immune cells [6]. Hsp70 is the molecular chaperone which can enhance the survival of cancer cells via binding several important signaling proteins and therefore eliciting protective functions [8]. Hsp70 can be secreted from cancer cells in a soluble form and/or being embedded in exosomes; irrespective of its form, the chaperone can penetrate viable cells and expose their antigens to lymphoid cells causing powerful anti-cancer effects [9, 10]. Calreticulin functions as a chaperone and Ca^2+^ buffer to assist in correct protein folding within the endoplasmic reticulum. The process of calreticulin translocation to the surface of tumor cells treated with certain therapeutic factors involves the secretion of ATP, Hsp70/90 and HMGB1 into the medium [11]. Importantly, the mechanisms governed by the three above alarmins in tumor and TME cells are linked to each other and involve other participants in the relapse process, like caspase-3 [5].

In order to explore these intermolecular linkages in more detail, we employed the model of Sulciner et al., in which the remnants of dying tumor cells activated the repopulation of sparse, rarely seeded acceptor cells. The great efficacy of the method was that specially prepared cell debris induced the growth of tumor cells injected into mice in minimal amounts; the authors demonstrated that Lewis lung carcinoma cells in the amount of 1–5 thousand treated by the debris fraction obtained from dying cells gave tumors with a size comparable to that of 1–2 million which is usually needed for tumor formation [12].

To study the role of extracellular Hsp70 in repopulation, we employed the conditioned medium of dying cancer cells and found that the depletion of the protein with two chromatography methods was able to modulate the growth-stimulatory activity of the medium. Interestingly, the removal of Hsp70 alone did not affect proliferation rate; furthermore, we demonstrated for the first time that Hsp70 forms a tight complex with HMGB1 and promotes the growth-activating potency of the latter. Importantly, dissociation of the Hsp70-HMGB1 complex with Hsp70 inhibitors strongly attenuated the repopulation capacity of the alarmins on sparse populations of tumor cells, both in vitro and in vivo.

## Materials and methods

### Cells and manipulations

#### Cells

Human lung adenocarcinoma A549 and H1299 and human colorectal adenocarcinoma cells DLD1 were obtained from the Collection of Cell Lines of the Institute of Cytology RAS, Russia. Mouse colon carcinoma CT-26 cells were kindly provided by Prof. G. Multhoff (Technical University of München, Germany). A549, H1299 and DLD1 cells were cultivated in DMEM and CT-26 cells were grown in RPMI-1640 media supplemented with 10% heat inactivated fetal bovine serum (FBS) (HyClone, USA), 2 mM L-glutamine, 100 U/mL penicillin and 0.1 mg/mL streptomycin (PanEco, Russia) in a 5% CO_2_ atmosphere with 90% humidity. Viability was determined by 0.4% trypan blue exclusion.

The CT-26 luc and A549 luc cell lines were transduced with the luciferase gene using the pHIV-iRFP720-E2A-Luc [10] and pHIV-Luciferase vectors, respectively. The development of A549_shHsp70_ and DLD1_shHsp70_ cells was recently described [13].

#### Proteins and peptides

Human recombinant Hsp70 was purified from *Escherichia coli* cells transformed with pMSHsp70 plasmid and detoxified with the use of polymyxin B–agarose gel (Sigma, St.Loise, MO, USA) as described elsewhere [10].

Recombinant human HMGB1 was isolated from *E. coli* BL21 (DE3) Star™, transformed with pET24a(+)-T7-HMGB1-His tag plasmid. The induction of HMGB1 expression was performed by the addition of 0.1mM IPTG to LB growth media followed by agitation at 200 rpm at 25 °C for 4 h. The cells were collected by centrifugation at 3000 rpm for 20 min at 4 °C and then lysed using an ultrasonic system in lysozyme-containing buffer. HMGB1 was then purified using HisPur ™ Ni-NTA Resin (Thermo Scientific) according to the manufacturer`s protocol.

For PPI and pull-down assay Hsp70 and HMGB1 were biotinylated with EZ—Link® Sulfo-LC-Biotin (Thermo Scientific, USA) in accordance with the manufacturer’s instruction.

HBHP and HBHP-scr peptides were synthesized with biotin on N-terminus by NPF Verta (Saint Petersburg, Russia).

#### Cell viability and cell growth assays

Values were estimated using the MTT test and xCELLigence technique. First, the mouse colon carcinoma CT-26 cells were seeded into wells of 96-well plates at a concentration of 1.6 × 10^5^ cells/ml and a day later, 3 µM etoposide (Pharmachemy B.V., the Netherlands) was added to the fresh medium. After 24 h, the medium with etoposide was removed, the cells were washed twice with PBS and pure medium was added. To determine the cytotoxicity induced by the use of etoposide, CT-26 cells were incubated in etoposide-free medium for 24, 48 and 72 h and cell viability was measured by the MTT assay according to the protocol described previously [14]. Next, A549 and DLD1 cells were seeded into wells of 96-well plates at a concentration of 500 cells per well and were then incubated with conditioned cell media prepared as described below; after 10–14 days, cell number was estimated by MTT assay. When using the xCELLigence technique, which allows cell populations to be followed in real time, A549 and DLD1 cells were seeded into the wells of E-plates, after which conditioned media was added and cell growth was recorded for 200–300 h.

#### Cell media preparation

A549_wt_, A549_shHsp70_, DLD1_wt_ and DLD1_shHsp70_ were seeded on 10 cm^2^ Petri dishes (TTP, Switzerland); when the monolayer reached 75%, 100 µM etoposide (Pharmachemie BV, the Netherlands) or 100 µM oxaliplatin (Oncotec Pharma Produktion GmbH, Germany) was added. Eighteen hrs later, cell media with anticancer drugs were removed and the cells were carefully washed to remove drugs before fresh serum-free medium was added for the next 48 h. After this, the conditioned media (CM) were collected, centrifuged (4000 g, 15 min) and the supernatants were transferred to clean tubes and kept at -80 °C before use.

To remove Hsp70 from the conditioned medium of A549 or DLD1 cells treated with etoposide (Eto-CM) or with oxaliplatin (Oxa-CM), 50 µl of ATP-agarose (Sigma, USA) in the presence of 2mM MgCl_2_ or 10 µl of biotinylated home-made antiHsp70 rabbit polyclonal antibody [10] following Neutravidin-Sepharose (50 µl) (Thermo Scientific, USA) were used. For HMGB1 removal, HBHP-peptide with the sequence HMSKPVQ that can directly bind HMGB1 [14] and scramble HBHP-scr peptide with the PMQSKHV sequence were used. Finally, aiming to destroy Hsp70-HMGB1 complexes, we used CM from A549 cells treated with 100 µM etoposide and 10 µM JG-98 (MedChemExpress, USA).

#### Colony formation assay

A549 or DLD1 cells were seeded in the wells of a six-well plate at a concentration of 250 cells/ml. After 18 h, the culture medium was changed to conditioned Oxa-CM or Eto-CM derived from A549_wt_, A549_shHsp70_, DLD1_wt_, DLD1_shHsp70_ and Oxa-CM or Eto-CM cells after immuno- or ATP precipitation. Cells were incubated for 10 days in 5% CO_2_ at 37 °C. Furthermore, grown colonies were fixed with 10% formaldehyde and stained with 0.01% crystal violet. The plate was dried and scanned using the ChemiDoc system (Bio-Rad, USA).

#### Immunohistochemistry

A549 cells were seeded to cover glasses and 24 h later were treated with 100 µM etoposide for 2 or 4 h. Cells were fixed with 4% paraformaldehyde, permeabilized with 0.1% Triton X-100 and stained with monoclonal mouse anti-Hsp70 antibody (Clone 3B5) followed by anti-mouse antibody labeled with Alexa555 (ThermoFisher, USA), or rabbit anti-HMGB1 antibody (Abcam, UK) followed by anti-rabbit antibody labeled with Alexa488 (ThermoFisher, USA). Nuclei were stained with 40,6-diamidino-2-phenolindole dihydrochloride (DAPI). Fluorescence images were captured by an Olympus FV3000 confocal microscope and analyzed with cellSens software.

#### Animals

All in vivo experimental protocols were approved by the licensing committee of the Institute of Cytology of the Russian Academy of Sciences (Identification number F18-00380). All methods were carried out in accordance with relevant guidelines and regulations and are reported in accordance with ARRIVE guidelines.

Male BALB/c (for subcutaneous CT26 tumor formation) and female BALB/c nude mice (for subcutaneous A549 tumor formation) used in this study were purchased from National Research Lobachevsky State University of Nizhny Novgorod (Russia). To determine which factor is able to stimulate the growth of a small cancer cell population, we first injected mice with a mixture of 10^6^ CT-26_wt_ cells treated with 3µM etoposide for 24 h and then washed out the drug, mixed samples with 2 × 10^4^ intact CT-26_luc_ and injected this subcutaneously into Balb/c mice that were randomly divided into two groups, each containing 10 animals. The control group received 2 × 10^4^ CT-26_luc_. Tumor growth was estimated 28 days later based on bioluminescence detection.

**Fig. 1:**
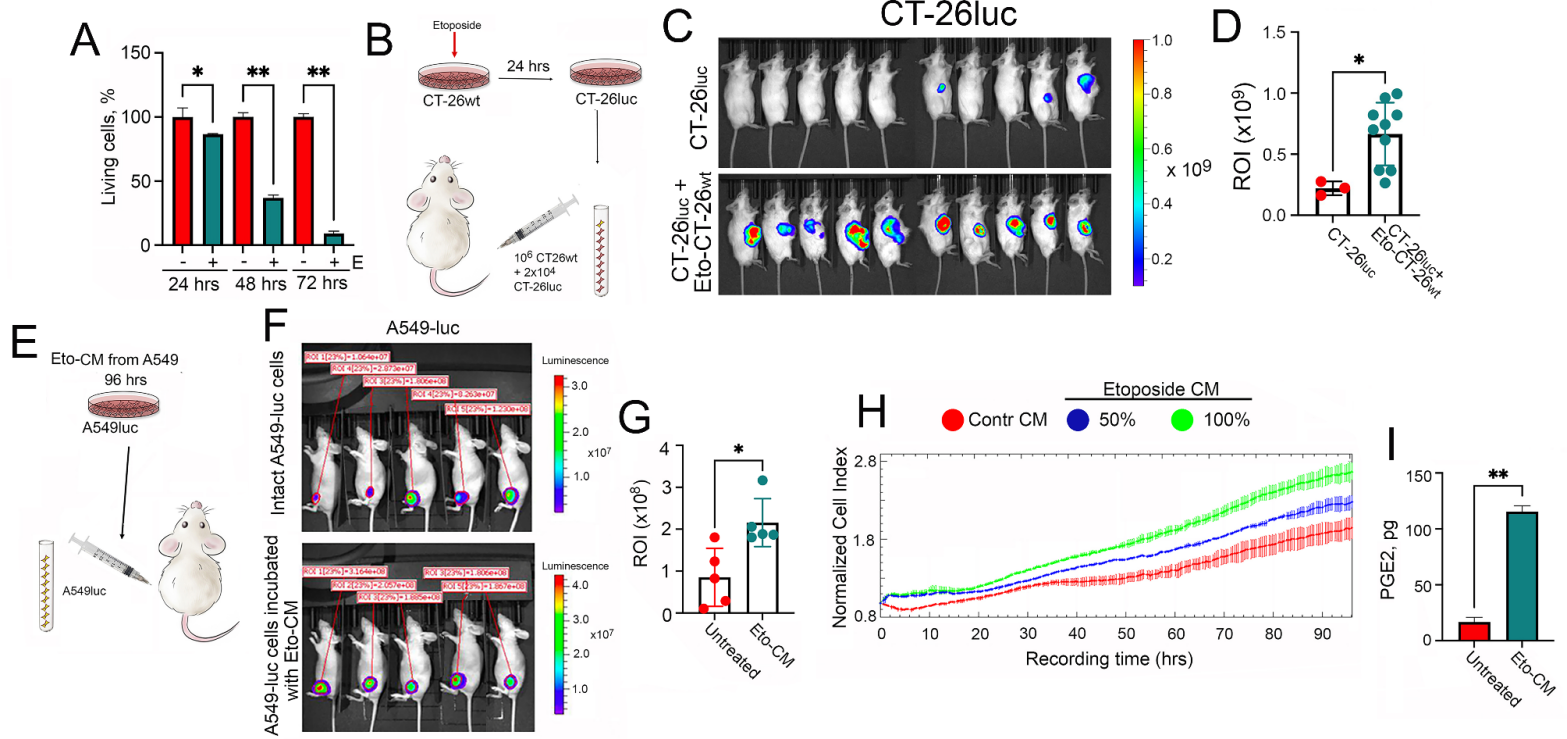
The factor stimulating the growth of small tumor cells population is found in the conditioned medium (CM) from dying tumor cells following chemotherapy. **(A)** Tumor cells treated with etoposide continued to die even in the absence of a chemotherapeutic drug. Mouse colon carcinoma CT-26 cells were treated with 3 µM etoposide for 24 h, then cells were washed and allowed to be in culture. Cell viability was evaluated each 24 h with aid of MTT assay. * p < 0.05, **p < 0,001. **(B)** Scheme of in vivo experiments with CT-26 cells: CT-26wt cells were treated with 3µM etoposide for 24 h, then were washed out and mixed with CT-26luc in a ratio of 106:2 × 104 and subcutaneously inoculated to Balb/c mice. Other mice groups received either 2 × 104 CT-luc cells or 106 dying CT-26wt (data not shown) cells. **(C)** CT-26luc tumors were evaluated with the aid of IVIS Spectrum imaging system on day 28. **(D)** Luminenscence count of tumor lesions from (C), *p < 0.05. **(E)** Scheme of in vivo experiments with A549 cells: A549 cells were treated with 100µM etoposide for 18 h, then washed out and for the next 48 h were kept without drug. Then, CM from these cells was collected and added to naive A549-luc cells for 96 h. Naive A549luc and A549luc were subcutaneously injected to Balb/c Nude mice. **(F)** A549-luc tumor growth from naive A549-luc and A549-luc cells incubated with CM was estimated using IVIS Spectrum imaging system on day 28. **(G)** Luminescence count of tumor lesions from D. *p < 0.05. **(H)** A549wt cells were seeded into wells of E-plates at a concentration of 500 cells per well and were allowed to grow in CM from untreated A549 cells, or with full (100%) or half-diluted (50%) Eto-CM. Recording on xCELLigence equipment lasted 180 h. **(I)** Data of PGE2 measurement in the CM of untreated A549 cells and Eto-CM. ** p < 0.001

Next, we used CM from A549_wt_ cells treated with etoposide as described above and incubated A549_luc_ cells with a mixture of Eto-CM and normal cell media at a ratio of 1:1 for 4 days. A549_luc_ cells were collected and injected into Balb/c nude mice at a dose of 2.5 × 10^4^ cells in Matrigel (Corning Incorporated, USA) (see Fig. 1A). Tumor progression was estimated 28 days later based on bioluminescence detection.

To study the therapeutic potency of JG-98, we incubated A549_luc_ cells with Eto-CM and Eto + JG98-CM from A549_luc_ cells (in addition to etoposide at a concentration of 100 µM, JG98 at a concentration of 10 µM was used) for seven days before being subcutaneously inoculated into Balb/c nude mice. Mice were divided into 3 groups, with 10 animals in each (Untreated, Eto-CM, Eto + JG98-CM). Every group received 2.5 × 10^4^ A549_luc_ cells. Tumor growth was estimated 41 days later after inoculation based on bioluminescence detection.

In all in vivo experiments, tumor cells suspended in the mixture of Matrigel (Corning Incorporated, USA) and DMEM (1:1) were injected subcutaneously. Tumor progression was estimated by the magnitude of its bioluminescence. For this purpose, mice were injected with 3 mg luciferin (Thermo Scientific, USA) dissolved in sterile DPBS. Then, mice were subjected to inhalation anesthesia with Aerrane (Baxter Healthcare Corporation, USA). Luminescence detection was performed with the aid of an IVIS Spectrum in vivo Imaging System (PerkinElmer, USA) in the mode of automatic selection of the signal accumulation time.

### Gel electrophoresis and western blotting

Eluates from samples precipitated with specific anti-Hsp70 rabbit polyclonal antibodies followed by ProteinG-Sepharose (Thermo Scientific, USA) precipitation and by ATP-agarose from CM of untreated A549 cells, Eto-CM and Oxa-CM, were supplied for SDS-electrophoresis in 12% polyacrylamide gel and stained with silver staining according to the previously described protocol [15]. These eluates and unbound fractions were also used for the co-precipitation assay. Proteins were transferred to PVDF membranes (Thermo Scientific, USA) using the Trans-Blot Turbo Transfer System (BioRad, USA). The membranes were probed with polyclonal rabbit antibodies against HMGB1 (Abcam, UK) and then with monoclonal rat antibodies against Hsp70 (Clone 3B5) followed by secondary horseradish peroxidase-conjugated anti-rabbit (Abcam, UK) and anti-rat antibodies (Thermo Scientific, USA). Chemiluminescence detection of the protein bands was performed with Clarity Western ECL Blotting Substrate and ChemiDoc MP equipment (both Bio-Rad, USA). Eluates obtained with HBHP_bio_ peptide were also electrophoresed in 11% PAGE, transferred to a PVDF membrane and then the membrane was probed with antibodies against HMGB1 and Hsp70. To measure autophagy levels, cell lysates were collected from A549 tumor cells pre-treated with the indicated CMs. Then, protein concentrations were measured using the Bradford assay and 30 µg of total protein was subjected to electrophoresis in a 15% polyacrylamide gel, before being transferred to PVDF membrane and probed using rabbit polyclonal antibodies against LC3 A/B (Cell signaling, USA), ATG5 (Cell signaling, USA), p62 (Abcam, UK) and rat antibodies against Tubulin (Abcam, UK). Next, membranes were incubated with the respective aforementioned secondary antibodies and the chemiluminescence of protein bands was detected.

### PGE2 production assay

The indicated CMs were collected as previously described and the level of PGE2 in the conditioned media was measured using a human PGE2 immunoenzyme assay kit (BlueGene Biotech, China), according to the manufacturer’s protocol.

### Real-time PCR

RNA was isolated using TRIziol (Thermo Fisher Scientific, USA) and converted to DNA using the MMV RT kit (OOO Evrogen, Russia) according to the manufacturer’s protocol. All RT-PCR studies were performed using the CFX96 Real-Time detection system (Bio-Rad, USA). Gene expression in the samples was analyzed using the qPCRmix-HS SYBR kit (OOO Evrogen, Russia) according to the manufacturer’s instructions. The authenticity of the amplicon was confirmed by analysis of the melting curve. Sequences of primers used in the study are inducated in Table 1.

**Table 1:** Sequences of primers used in the study

Aurka	Forward	5`-GCTGGAGAGCTTAAAATTGCAG-3`
	Reverse	5`-TTTTGTAGGTCTCTTGGTATGTG-3`
mcm10	Forward	5`-ACCACCAAGACCAAAACTGAGT-3`
	Reverse	5` -GATGTCCTGAGGGTCCTTTTGT-3`
ki-67	Forward	5`-TCCTAGGAAAACTCCAGTTGCC-3`
	Reverse	5`-AGACACTCTCTTTGAAGGCAGG-3`
β-actin	Forward	5`-CCATCATGAAGTGTGACGTGC-3`
	Reverse	5`-GTCCGCCTAGAAGCATTTGCG-3`

### Proteomic analysis

For shotgun proteomics, we performed “in-beads” digestion. Beads were mixed with 50 µl of 8 M urea/50 mM ammonium bicarbonate (Sigma Aldrich, St. Louis, MO, USA). Then cysteine alkylation was performed by (1) incubation with 5 mM DTT (Sigma Aldrich, St. Louis, MO, USA) for 1 h at 37 °C and (2) incubation in 15 mM iodoacetamide for 30 min in the dark at room temperature (Sigma Aldrich, St. Louis, MO, USA). Finally, 5 mM DTT was added to quench iodoacetamide and the samples were diluted with seven volumes of 50 mM ammonium bicarbonate. For tryptic digestion, samples were incubated for 14 h at 37 °C with 250 ng of proteomics grade Trypsin (“Trypsin Gold”, Promega, Madison, WI, USA). For tryptic peptide extraction, samples were centrifuged for 5 min at 5000 g and supernatant was collected. Then, we added 100 µl of formic acid (Sigma Aldrich, St. Louis, MO, USA) to beads, vortexed them and centrifuged for 5 min at 5000 g. The supernatant was collected.

Tryptic peptides were desalted by self-made stage tips prepared according to Matamoros et al.: polypropylene pipette tips (200 µL; SSIbio, USA) were filled with four layers of C18 reversed-phase excised from Empore 3 M C18 extraction disks. The desalted peptides were evaporated in a Labconco Centrivap Centrifugal Concentrator (Labconco, USA) and stored at -20 °C prior to analysis.

Desalted peptides were dissolved in water/0.1% formic acid for further LC-MS/MS with ion mobility in TimsToF Pro mass spectrometer (Bruker Daltonics, Bremen, Germany) with nanoElute UHPLC system (Bruker Daltonics, Bremen, Germany).

UHPLC was performed in a two-column separation mode with Acclaim™ PepMap™ 5 mm Trap Cartridge (Thermo Fisher Scientific, Waltham, MA, USA) and Bruker Ten separation column (C18 ReproSil AQ, 100 mm × 0.75 mm, 1.9 μm, 120 A; Bruker Daltonics, Bremen, Germany) in gradient mode with 500 nL/min flow rate and separation column temperature of 50 °C. Phase A was water/0.1% formic acid, while phase B was acetonitrile/0.1% formic acid. The gradient was from 2 to 30% phase B for 16 min, then to 38% of phase B for 5 min and 95% for 3 min, with a subsequent wash with 95% phase B for 6 min. The column was equilibrated with 4 column volumes before each sample. CaptiveSpray ion source was used for electrospray ionization with a 1600 V of capillary voltage, 3 l/min N2 flow and 180 °C source temperature. The mass spectrometry acquisition was performed in the automatic DDA PASEF mode with a 0.5 s cycle in positive polarity with the fragmentation of ions with at least two charges in m/z range from 100 to 1700 and ion mobility range from 0.85 to 1.30 1/K0. Each sample was analyzed four times with blanks.

Protein identification was performed in Peaks Xpro software (a license granted to St. Petersburg State University; Bioinformatics Solutions Inc., Waterloo, ON, Canada) using the human protein SwissProt database (https://www.uniprot.org/; accessed on 17 October 2021; organism: Human [9606]; uploaded on 2 March 2021; 20,394 sequences) and protein contaminants database CRAP (https://www.thegpm.org/crap/; version of 4 March 2019; accessed on 17 October 2021). The search parameters were as follows: parent mass error tolerance 10 ppm and fragment mass error tolerance 0.05 ppm, protein and peptide FDR less than 0.1%, two possible missed cleavage sites, proteins with at least two unique peptides; these were all included for further analysis. Cysteine carbamidomethylation was set as a fixed modification. Methionine oxidation, the acetylation of the protein N-term, asparagine and glutamine deamidation were set as variable modifications.

Label-free quantitative analysis was performed in Peaks Xpro software with proteins having at least 2 peptides, with a peptide count of at least 2 and detected in at least 3 of the 4 samples per group. Qualitative analysis was performed in R (version 4.1.1). The proteins were assigned to a biological group if they were identified in three of the four replicates. Functional annotation was performed in DAVID Bioinformatics Resources 6.8 Functional annotation tool (https://david.ncifcrf.gov/, accessed 09.01.2022).

#### FRET

Förster resonance energy transfer (FRET) was used to analyze the interaction between Hsp70 and HMGB1. For this purpose, purified human recombinant Hsp70 was labeled with Alexa488 fluorescent dye (Thermo Fisher Scientific, USA) and recombinant HMGB1 was labeled with Alexa555 fluorescent dye (Thermo Fisher Scientific, USA). Labeling procedure was produced according to manufacturer’s protocols. Experimental mixtures containing 50 µg/ml of Hsp70-488 and 10, 20 or 50 µg/ml of HMGB1-555 in 200 µL of TBS were incubated for 1 h, then the fluorescence emission spectrum of each sample was determined using a Varioscan LUX device (Thermo Fisher Scientific, USA). The fluorescence emission spectrum was detected from 515 to 620 nm at the excitation wavelength of 490 nm. Then fluorescence emission at 568 nm at the excitation wavelength of 490 nm was separately measured. For all data, the final fluorescent signals were obtained by subtracting the fluorescent signals with the background noise from а blank well (200 µL of TBS containing proteins without fluorescence labels). The experiments were repeated four times and the average value of fluorescence was taken at each specific condition.

#### Protein-protein Interaction assay (PPI)

Capture antibody to HMGB1 was placed in wells of 96-well plates and, after blocking with 2 mg/ml BSA in 20 mM borate buffer, pH 8.0, pure HMGB1 at a concentration of 1 µg/ml or 10 µg/ml was loaded to wells to 2 h; after washing, Hsp70 at concentrations of 0.1 µg/ml, 1.0 µg/ml or 10 µg/ml was added for 3 h. Next, detecting biotinylated anti-Hsp70 antibody was followed by HRP-conjugated Avidin (Merk, USA). In experiments aiming to find available low molecular-weight Hsp70-HMGB1 uncouplers such as MKT-007, PES and JG-98, compounds were added and pre-incubated with Hsp70 at a concentration of 10 µM for 1 h. After washing with TBS with 0.05% Tween 20 (Sigma, USA), biotinylated anti-Hsp70 antibodies were applied followed by HRP-conjugated Avidin. The optical density at 450 nm was measured using a Varioscan LUX microplate reader (Thermo Fisher, USA). Hsp70-HMGB1 complexes in Eto-CM and Eto-CL were revealed with the aid of PPI assay in an opposite design, on the bottom of wells of the 96-plate capture anti-Hsp70 antibodies were placed and after blocking with 2 mg/ml BSA, 350 µg/ml of A549 cells lysate from untreated treated cells with etoposide for 2, 4 and 8 h, or CM from the same cells, was applied for 3 h. After washing, detecting biotinylated anti-HMGB1 antibodies was added followed, by HRP-conjugated Avidin. The same protocol was used for Hsp70-HMGB1 complexes analysis in Eto-CM of A549 cells treated also with 25 µM PES or 25 µM JG-98.

#### Pull-down assay

Hsp70 and HMGB1 were biotinylated and then 10 µg of each protein was added to the CM of A549 cells treated with 100 µM etoposide or 100 µM oxaliplatin and incubated for 4 h with rotation. Then, 50 µl Neutravidin-Sepharose was added for 1 h. The beads were washed with PBS buffer with 0.05% Tween 20, incubated in boiled solution for SDS-polyacrylamide gel electrophoresis and resulting gel was subjected to western blotting with the appropriate antibody recognizing either Hsp70 or HMGB1.

#### Statistics

Data are reported as the mean ± standard error of the mean and represent the data of at least three independent experiments. Quantitative analysis was performed with the use of Graph Pad Prism 9.4.1 (Graph Pad Software Inc, San Diego, CA, USA). One-way ANOVA test followed by Tukey’s multiple comparisons test was used. Differences were considered to be statistically significant at p < 0.05.

## Results

### The growth-stimulating factor of tiny tumor cell populations occurs outside cells

One of the factors capable of inducing the repopulation of tumor cells remaining after anti-cancer therapy is the fragments of dying cells, their debris [16]. We probed two ways to check whether the material of dying tumor cells is able to stimulate the repopulation in sparse cell populations. First, to obtain tumor cell debris, we employed the protocol of Huang et al. [17] and substituted radiotherapy, which is often employed to kill the primary tumor, with chemotherapy using etoposide at cytotoxic concentrations. Mouse colon cancer CT-26 cells have been treated with 3 µM etoposide for 24 h and after washing out the drug, CT-26 cells were left to die for the next 72 h (Fig. 1A). Even in the absence of the drug, cells continued to die; within 48 h, the percentage of survived cells decreased to 35% and by 72 h it decreased to less than 10% (Fig. 1A). Next, 10^6^ CT-26_wt_ cells incubated with etoposide for 24 h were employed as the source of the debris and then mixed with 2 × 10^4^ CT-26_luc_ cells expressing luciferase before being injected subcutaneously into syngeneic Balb/c mice (see the scheme in Fig. 1B). As a control, mice injected with 2 × 10^4^ CT-26_luc_ cells alone were used. Twenty eight days later, we observed five growing tumors in mice injected with mixtures of dying and alive cells (10 animals in each group) (Fig. 1C, lower panel); in the control group injected with CT-26_luc_ alone, there were only three tumors (Fig. 1C, upper panel). These data proved that chemotherapy resulted in massive cell death effectively induce the growth of sparse populations of cancer cells.

Next, we employed another protocol to initiate the dying cell-induced repopulation via using human lung carcinoma A549_wt_ cells and the same type of treatment as above. Treatment with etoposide lasted for 18 h, after which the drug was removed and Eto-CM was collected after 48 h of incubating cells and then transferred to cells expressing luciferase (A549_luc_) for next four days (the scheme in Fig. 1D). These cells and intact A549_luc_ cells were subcutaneously inoculated to Balb/c nude mice. As a result, tumors grew in all mice, but in the group of mice that received the pre-incubated tumor cells with Eto-CM, the tumor size was 2.5-fold larger, suggesting that the Eto-CM contained substances required for effective repopulation in vivo (Fig. 1E, F). To prove the existence of relapse-inducing factors in vitro, we collected CM from A549 cells treated with etoposide and added either full CM (100%) or CM half-diluted with fresh medium (50%) to A549 cells seeded into the wells of E-plates at a concentration of 500 cells per well and cell growth was monitored in real-time using xCELLigence equipment. As proposed, the cell index for untreated cells was 2.02, for the cells incubated with 50% of conditioned medium it was 2.24, while for the cells incubated with full CM it was 2.42, indicating that the factors that stimulated tumor cell growth exists in the conditioned medium (Fig. 1G). Since it has been previously shown that the recurrence of tumor cells can be induced by the production and secretion of prostaglandin E_2_ (PGE2) [18], we measured the latter concentration in the supernatant of tumor cells following their incubation with Eto-CM for 48 h and found that PGE2 production and secretion levels were 7-fold higher following incubation of cells with Eto-CM compared to CM from untreated tumor cells (Fig. 1H).

In conclusion, CM collected from etoposide-treated, dying cells was capable of inducing proliferation in rearely seeded cancer cells imitating minimal residual disease [19] Mishra et al., 2023]

### Hsp70 is critical for tumor cells repopulation

Tumors of different histogenesis often exhibit elevated Hsp70 expression [8]. The protein is believed to constitute a basis for the chaperone mechanism and efficiently protects cancer cells by binding proteins responsible for the execution of apoptosis; moreover, Hsp70 has been found to release from tumor cells, playing the role of a DAMP [20].

**Fig. 2:**
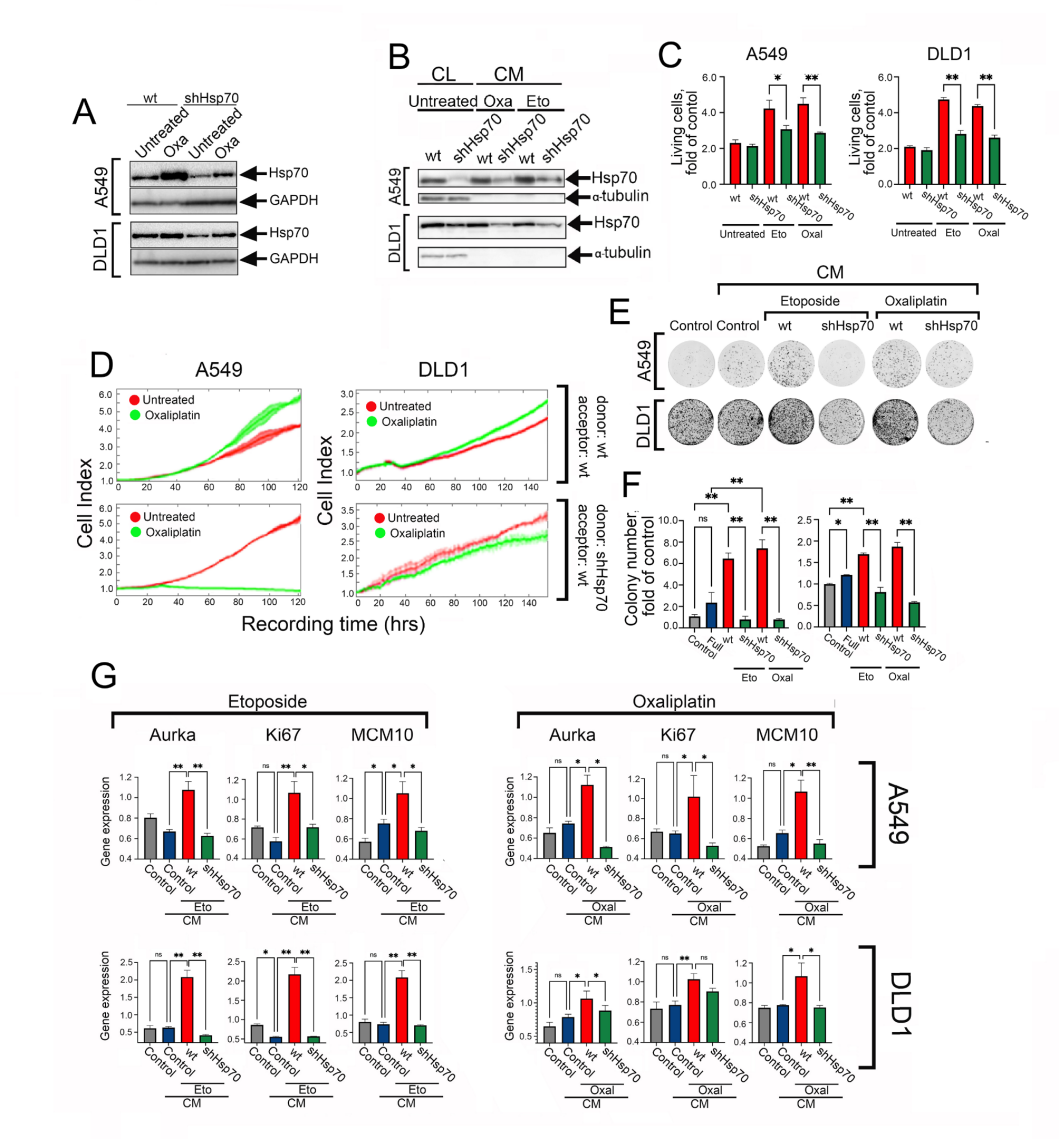
Hsp70 resencein CM increases the repopulation of tumor cells. (**A**) Western blotting of A549 and DLD1 cells with normal and reduced Hsp70 levels treated with 100µM oxaliplatin during 12 h. (**B**) Western blot of CL of A549 and DLD1 (wt and shHsp70) and CM from these cells after incubation with etoposide or oxaliplatin. (**C**) MTT assay data of A549 and DLD1 cells incubated in CM from wt or shHsp70 cells after etoposide or oxaliplatin treatment for 14 days. (**D**) Data of xCelligence experiment on A549 and DLD1 cell growth in presence of Eto-CM or Oxa-CM from wt and shHsp70 cells. (**E**) Colony formation of A549 and DLD1 cells in presence of Eto-CM or Oxa-CM from wt and shHsp70 cells. (**F**) Colonies intensity, presented in folds of control. (**G**) Repopulation marker genes expression in A549 and DLD1 cells in presence of Eto-CM or Oxa-CM from wt and shHsp70 cells

**Fig. 3:**
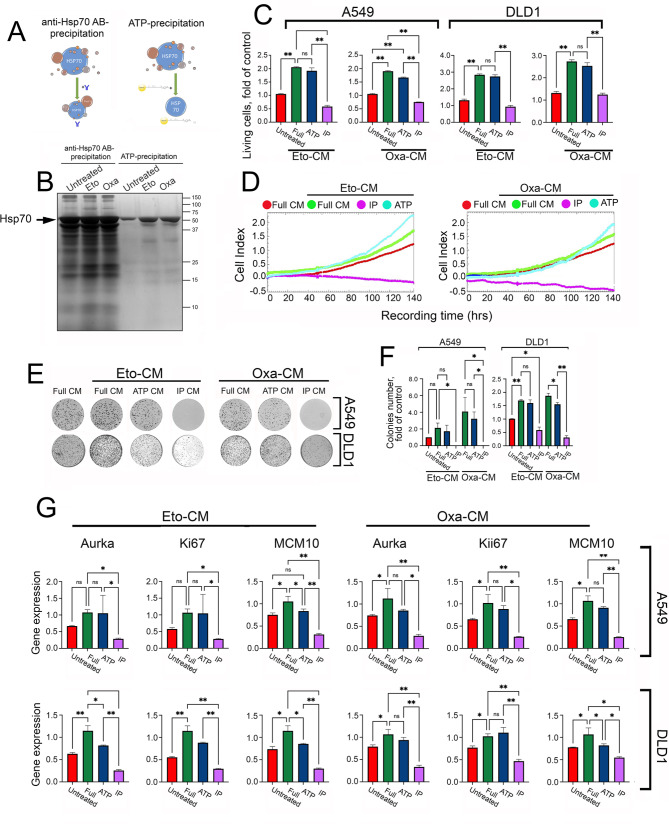
The repopulation-stimulating effects of CM is not exhibited by Hsp70 alone, but by Hsp70 together with some binding proteins. (**A**) A scheme demonstrating the difference in the composition of eluates during ATP precipitation and immunoprecipitation. (**B**) Ag-stained gel from CM from untreated A549 cells and from Eto-CM and Oxa-CM performed with specific antibodies and with ATP-agarose. (**C**) A549 and DLD1 cells were seeded into wells of 96-well plate at a concentration of 500 cells per well and CMs from untreated eponymous cells of Eto-CM and Oxa-CM depleted with ATP or IP were applied for 14 days, then cell growth was evaluated using MTT test. ** p < 0.001. (**D**) The A549 cells were treated as in (**C**) and their growth was evaluated with xCELLigence technique in real time. The results of one of many experiments are presented. Data for DLD1 cells, see the supplementary file Suppl (Fig. 2S). (**E**) Colony formation of A549 and DLD1 cells in presence of Eto-CM or Oxa-CM depleted with ATP or IP. (**F**) Colonies intensity, presented in folds of control. (**G**) Expression of repopulation marker genes in A549 and DLD1 cells in presence of Eto-CM or Oxa-CM depleted with ATP or IP

To check whether Hsp70 is implicated in tumor cell repopulation, we used lung carcinoma A549 cells with normal (A549_wt_) and reduced Hsp70 levels (A549_shHsp70_) and the similar pair of colon carcinoma DLD1 cells (DLD1_wt_ and DLD1_shHsp70_); in both sub-lines, the reduction of Hsp70 content was due to shRNA-mediated knockdown (Fig. 2A). The cells were subjected to deadly concentrations of two anti-cancer drugs, etoposide (Eto) and oxaliplatin (Oxa) and the level of Hsp70 was measured with the aid of western blotting. The immunoblotting results showed that the use of oxaliplatin caused an increase in Hsp70 content in tumor cells of both lines (Fig. 2A). Of note, a similar effect of anti-cancer drugs was observed earlier [21], so we confirmed this effect for two other cell lines. Next, we measured Hsp70 levels in CM after treating the cells of four sub-lines with etoposide and oxaliplatin and found that the level of Hsp70 in CMs from A549wt and DLD1wt cells was significantly higher than in media of cells with reduced expression of Hsp70 (Fig. 2B). Next, the conditioned media with distinct levels of Hsp70 were applied to naive A549_wt_ and DLD1_wt_ cells that were seeded in an extremely sparse mode to imitate in our experiments the cells remaining after chemotherapy. Fourteen days later, the number of growing cells was estimated using MTT test and we found that conditioned media of wt cells after treatment with Eto or Oxa increased A549 and DLD1 cell proliferation by 1.5- to 2-fold compared to the same cells grown in CM of untreated cells (Fig. 2C). These results were confirmed with the aid of the xCELLigence technique that allowed us to monitor the regrowth dynamics in real-time (Fig. 3D). In these experiments, we also used H1299 human lung carcinoma cells with normal and reduced Hsp70 levels and observed a similar effect of Oxa-CM on cell growth (Fig. 1S). Data of the colony formation assay also demonstrated the increased progression of naive cells treated with CMs containing elevated amounts of Hsp70 (Fig. 2E,F), indicating a proliferation-inducing effect of CMs with increased levels of Hsp70 on residual tumor cells. Additionally, we analyzed several markers known to indicate the progression of tumor cell growth such as Aurka (Aurora kinase A), Ki67 and MCM10, to estimate the level of tumor cell repopulations incubated in CMs with distinct contents of Hsp70. The activation of Aurka is necessary for the regulation of mitosis [22] and its expression is elevated in cancer tissues compared to normal ones [23]. Ki67 is a well-established proliferation marker [24] and MCM10 (minichromosome maintenance complex component 10) increased levels in lung cancer cells have been shown to correlate with recurrence and poor prognosis [25]. The analysis of mRNA levels of these markers in cells incubated with various CMs using real-time PCR showed that Eto-CM and Oxa-CM in both A549_wt_ and DLD1_wt_ cells considerably elevated Aurka, Ki67 and MCM10 expression levels, whereas incubation in CMs from shHsp70 cells had no effect on the expression of the analyzed genes (Fig. 2G), further proving a critical role of Hsp70 in stimulating the regrowth of sparse tumor cell populations in vitro.

### Does Hsp70 itself induce the repopulation?

To prove the particular role of Hsp70 in stimulating tumor growth in sparse cell populations, we removed the protein from Eto-CM and Oxa-CM before applying the latter media to cells. For this purpose, we employed two methods (i) using ATP-agarose or (ii) immunoprecipitation (IP) with a specific anti-Hsp70 antibody associated with protein G-Agarose. Chromatography on ATP-agarose is employed to isolate pure Hsp70 from the complex protein mixtures as we have previously demonstrated the method’s efficacy in pulling out Hsp70 from culture medium [26]. On the contrary, the immunoprecipitation with specific anti-Hsp70 antibodies does not interfere with the chaperone interactions with other cellular proteins (Fig. 3A). Hence, Eto-CM and Oxa-CM were collected as previously described and incubated with ATP-agarose or anti-Hsp70 antibodies attached to Protein G-Agarose. After washing, agarose slurries were subjected to electrophoresis and the resulting polyacrylamide gel was stained using silver dye method.

The Ag-stained gel showed that almost pure Hsp70 from untreated A549 cells-CM as well as from Eto-CM and Oxa-CM bound to ATP-Agarose, whereas IP probes contained many other polypeptides besides Hsp70, moreover, in the CM from Eto- and Oxa-treated cells, the number of the co-immunprecipitated proteins was markedly higher probably due to extensive cell death and release of bigger amounts of cell content (Fig. 3B).

Next, in order to study how the depletion of Hsp70 alone or its complexes with other proteins could affect the repopulation activity of CMs, we applied the Hsp70-depleted CMs on rarely seeded A549 and DLD1 cell populations; on the 14th day, cell growth activity was measured using MTT assay. Surprisingly, the depletion of Hsp70 alone with the use of ATP-Agarose did not affect cell repopulation in both cell lines; however, the immunoaffinity removal of Hsp70 complexes with other proteins resulted in a more than 3-fold decrease in the growth-stimulating activity (Fig. 3C). This reduction of repopulation in dynamics was more convincingly demonstrated using the xCELLigence technique, when depletion of Hsp70 from Eto-CM and Oxa-CM in complex with other polypeptides almost completely eradicated A549 cell growth, while Hsp70 depletion with ATP-agarose did not affect cell proliferation (Fig. 3D). Similar results were obtained when analyzing the response of DLD1 cells to Hsp70-depleted CMs (Suppl Fig. 2).

Colony formation assay also confirmed the loss of proliferation-stimulating properties of Eto-CM and Oxa-CM upon depletion of Hsp70-polypeptides complexes (Fig. 3E, F). The colony number of A549 cells incubated in immuno-depleted Eto-CM and Oxa-CM were equal to 0.019 ± 0.004 and 0.007 ± 0.002, respectively. When using the same CM, but depleted with ATP-agarose, the colony number was equal to 3.2 ± 0.4 and 1.7 ± 0.4, respectively, which was not significantly different from cells incubated in full Eto-CM or Oxa-CM. The withdrawal of Hsp70 by immune-depletion from Eto-CM and Oxa-CM collected from DLD1 cells also significantly abolished the proliferation-inducing competence, whereas ATP removal of Hsp70 had almost no effect on cell proliferation compared to cells incubated with full Eto-CM and Oxa-CM (Fig. 3E,F).

To confirm the effect of Hsp70 depletion on cell regrowth, we analyzed the expression of the repopulation genes markers, Aurka, Ki67 and MCM10 [22–24]. Consistently, ATP depletion of Hsp70 did not affect the expression of the above genes, while the removal of Hsp70 in putative complex with other proteins remarkedly reduced the expression of those proliferation-specific markers (Fig. 3G). When combined, these data indicate that Hsp70 per se is not essential for tumor cell regrowth, while its removal from the conditioned medium with coupled polypeptides strongly inhibits all attributes of repopulation, suggesting that the chaperone binds and supports the activity of factors that stimulate the proliferation of minimally residual tumor cells. Notably, removal of Hsc70, protein with structure and sequence close to Hsp70 but seemingly having distinct peptide-binding activity from the conditioned medium of A549 cells treated with etoposide, did not cause the loss of repopulation progress (Suppl. Figure 4).

### Hsp70 forms a complex with another DAMP, HMGB1

**Fig. 4:**
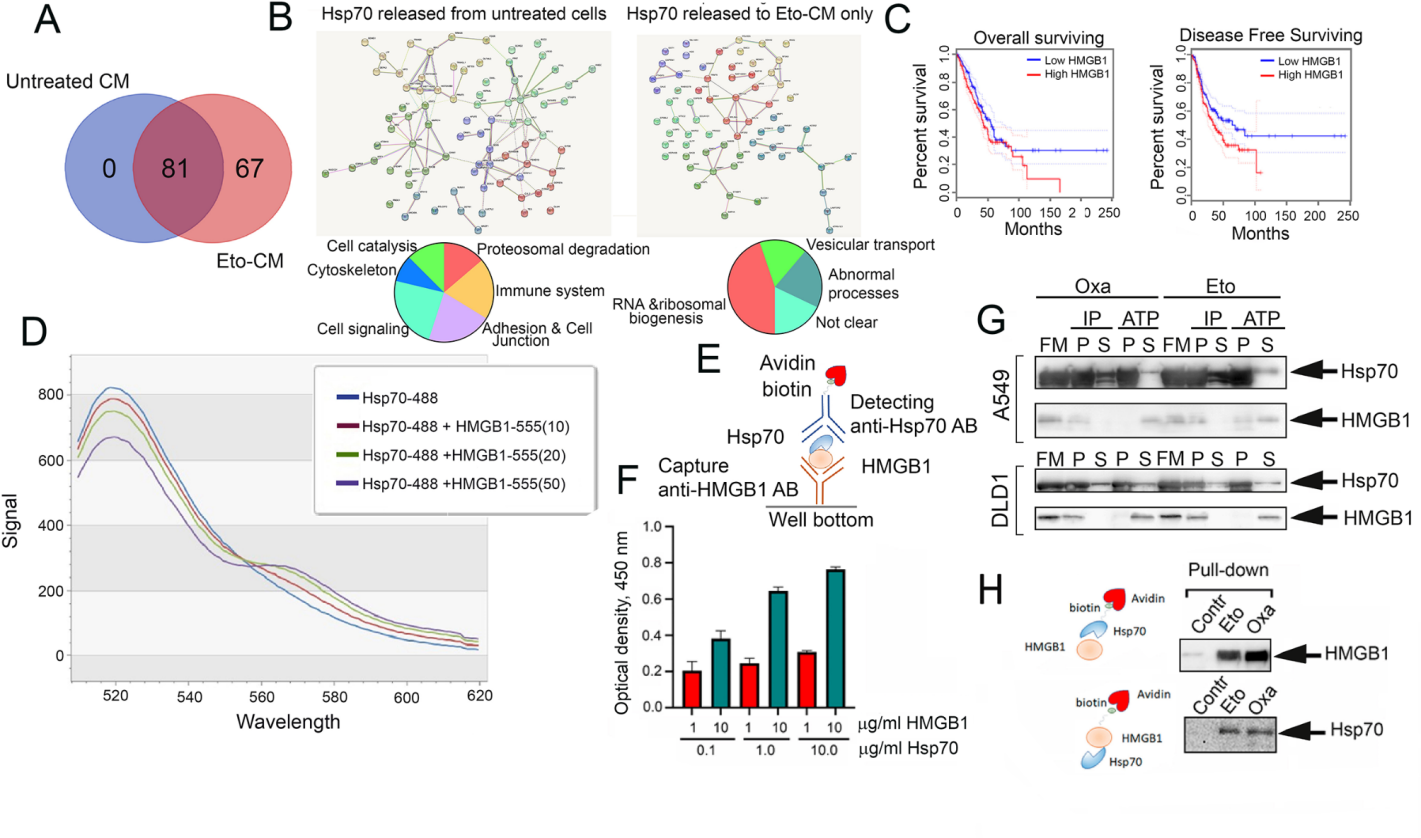
Hsp70 forms a complex with another DAMP, HMGB1. (**A**) Venn diagram of Hsp70-binding protein precipitated with anti-Hsp70 antibodies from untreated A549 cells and treated with etoposide. (**B**) Interactome of Hsp70 released from untreated A549 cells (left panel) divided to six clusters; in right panel: interactome of Hsp70 released from etoposide-treated A549 cells. Among Hsp70-binding proteins found in Eto-CM but not in CM from untreated cells, HMGB1 was found. (**C**) The retrospective analysis of overall survival and disease-free survival in lung and colon cancer patients carrying tumors with high and low levels of HMGB1. (**D**) FRET analysis of purified Hsp70-HMGB1 interaction. Representative emission spectra detected at 516–600 nm, excitation at 488 nm. The HMGB1-555 concentration is indicated in µg/ml in brackets. The Hsp70-488 concentration was 50 µg/ml. (**E**) Scheme of Protein-Protein Interaction assay (PPI). (**F**) results of PPI using pure HMGB1 and Hsp70. Antibody to HMGB1 (capture AB) was placed on the bottom of 96-well plates and then HMGB1 at a concentration of 1 or 10 µg/ml was added. After blocking, Hsp70 at concentrations of 0.1, 1.0 or 10 µg/ml was applied. Hsp70 in complex was identified with biotinylated anti-Hsp70 antibody (see (**E**)). (**G**) Western blotting of ATP or IP eluates (P) and unbound fraction (S) from Eto-CM or Oxa-CM from A549 cells. The membranes were probed with anti-HMGB1 and anti-Hsp70 antibodies. (**H**) Data of pull-down assay with biotinylated Hsp70 or biotinylated HMGB1 in Eto-CM and Oxa-CM of A549 cells

In order to identify the Hsp70-binding protein capable of promoting the repopulation, we performed a proteomic analysis of the probes obtained after IP with anti-Hsp70 antibodies from the CM of untreated A549 cells and those treated with etoposide. In CM of untreated cells, we found 81 Hsp70-binding proteins; the same proteins were found to be bound with Hsp70 in Eto-CM, while we also found in the latter 67 additional polypeptides (Fig. 4A). The analysis of the interactome of Hsp70 performed with the aid of STRING data base, demonstrated that in CM of untreated cells the chaperone was found to bind proteins involved in proteasomal degradation, regulation of immune system, process of cell adhesion, cell signaling, cytoskeleton and cell catalysis. Among the proteins found in the interactome of Hsp70 released from Eto-treated cells 30 polypeptides were found to be implied in the process of translation (Fig. 4B). Notably, among those 67 proteins, we found HMGB1 (high mobility group protein B1) (Fig. 3S) which, in addition to its role as a nuclear non-histone protein is involved in the regulation of transcription and chromatin remodeling, has a critical role as a DAMP that can be secreted outside a cell and participate in the activation of tumor cell repopulation [5, 27]. The latter function of HMGB1 allowed us to propose it as a key player in complex with Hsp70. Moreover, a retrospective analysis performed using GEPTA data base showed a dependence of overall and disease-free survival on HMGB1 level in patients with lung cancer (Fig. 4C).

Therefore, to prove whether Hsp70 forms a complex with HMGB1, we used Fönster resonant energy transfer (FRET). Using, Alexa488 conjugated Hsp70 as a fluorophore-donor, and Alexa555 conjugated HMGB1 as a fluorophore-acceptor, the latter applied in various concentrations, we obtained the emission spectrum in the range of 516–600 nm at an excitation wavelength 490 nm. As a result, a progressive increase in FRET signal at 568 nm was detected, which directly correlated with HMGB1 concentration (Fig. 4D), indicating that Hsp70 can indeed form a complex with HMGB1.

To further validate the FRET data, we performed a Protein-Protein Interaction assay (PPI assay) which was established as explicated in Fig. 4E (Fig. 4E); its results demonstrated that HMGB1 binds pure Hsp70 in a dose-dependent manner (Fig. 4F). In addition, the western blotting analysis of precipitates obtained after the incubation of Eto-CM or Oxa-CM with anti-Hsp70 antibody or ATP-agarose revealed that (i) both Hsp70 and HMGB1 are present in full CM of A549 cells treated with etoposide or oxaliplatin; (ii) in immunoprecipitates obtained with anti-Hsp70 antibodies (P), we found a high amount of HMGB1, while the amount of HMGB1 in the unbound fraction (S) was in a trace quantity; and (iii) after ATP-precipitation, most HMGB1 was found in the unbound fraction (S) (Fig. 4G). Lastly, the data of pull-down assay in which biotinylated Hsp70 or HMGB1 were employed to drag their partner polypeptides from Eto-CM or Oxa-CM also showed the complex persisting in culture media able to activate regrowth of A549 minimal population Fig. 4G).

### The Hsp70-HMGB1 complex is critical for tumor cells repopulation, neither Hsp70 nor HMGB1 alone

The next question, which could not be done without an answer, was whether HMGB1 alone could promote the regrowth of tiny tumor cell populations. To this end, we used the heptameric HBHP peptide with the sequence HMSKPVQ that directly binds HMGB1 [28], to remove HMGB1 or the Hsp70-HMGB1 complex from Eto-CM of A549 cells. As a scrambled peptide, we used the heptameric PMQSKHV peptide (HBHP-scr) proposed by the same authors [28]. The peptides were synthesized in biotinylated form, that allowed us to pull out HMGB1 alone or in complex with other proteins from Eto-CM.

**Fig. 5:**
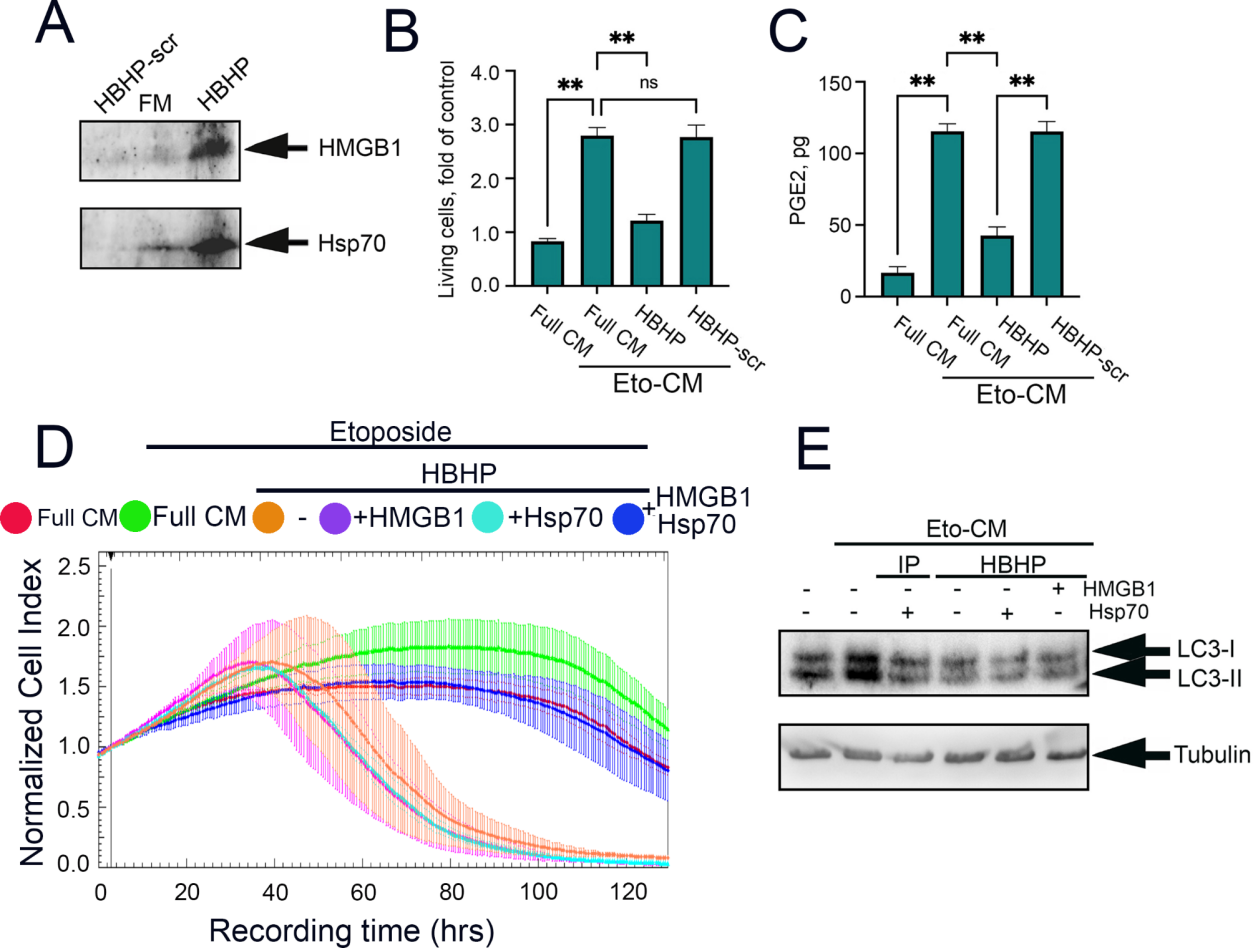
To stimulate tumor cell repopulation, Hsp70-HMGB1 complex is needed, not Hsp70 or HMGB1 separately. (**A**) Western blot of HBHP- and HMGB1-scr peptide eluates from Eto-CM of A549 cells. (**B**) A549 cells were seeded into wells of 96-well plates at a low concentration and then were incubated in Eto-CM, full and depleted with HBHP-peptide as well as HBHP-scr peptide. Cell growth was evaluated with MTT assay on day 14. **p < 0.001.(**C**) Level of PGE2 expression in in full or depleted Eto-CM measured with use of human PGE2 immunoenzyme assay kit; (**D**) HMGB1 was depleted from CM of untreated A549 cells and Eto-CM and then purified HMGB1 or purified Hsp70, or their complex were added and these media and were applied to A549 seeded to E-plates at a low concentration. Cell growth in real time was estimated using the xCELLigence technique. (**E**) A549 cells were incubated with the same CMs as in (**C**) and (**D**) for 24 h and then cells were lysed and used for western blotting with antibodies against autophagy marker LC3 I-II

First, to confirm that the HBHP peptide is able to remove HMGB1 from CM of cells treated with anti-cancer drugs, we precipitated HBHP and HBHP-scr using Neutravidin Agarose beads. Indeed, the results of the affinity purification demonstrated that the HBHP peptide removed HMGB1 in complex with Hsp70, while the HBHP-scr was not able to bind HMGB1 (Fig. 5A).

Next, we applied the Eto-CM depleted with HBHP peptide or HBHP-scr peptide to A549 cells and ten days later, measured cell growth using the MTT assay. The results showed that the application of full Eto-CM increased cell proliferation in A549 cells up to 278.0 ± 5.8% of the control cells; the application of HBHP-scr did not slow cell growth, but the use of HBHP peptide reduced cell proliferation down to 119.8 ± 6.0%, which was 2.3-fold lower (Fig. 5B). Additionally, to test whether cell growth inhibition mediated by HMGB1 depletion was due to reduced prostaglandin signaling, we measured the latter levels and found that PGE2 production was significantly downregulated upon using the specific peptide as bait while HBHP-scr did not affect PGE2 production by repopulating cells (Fig. 5C).

Then, using the xCELLigence technique, we evaluated the cell growth dynamics of A549 cells in Eto-CM depleted with HBHP-peptide. In these experiments, pure proteins, human recombinant Hsp70 or human recombinant HMGB1 or their complex. were introduced into Eto-CM that was preliminarily depleted of their complex. Consequently, the addition of pure HMGB1 did not affect cell growth dynamics, irrespective of whether it was introduced in the CM of untreated cells or the Eto-CM depleted with HBHP peptide (Fig. 5D and Suppl Fig. 5, upper panel). Similarly, the addition of pure Hsp70 had no effect on growth of A549 cells incubated in HBHP-depleted Eto-CM (Fig. 5D and Suppl Fig. 5, lower panel).

Since cancer relapse is predominantly associated with the activation of pro-survival autophagy and HMGB1 has been demonstrated to regulate this process [29], we analyzed the most abundant marker of autophagy, LC3 I/II [30, 31]. Western blotting data showed that LC3 II expression was markedly upregulated in cells incubated in Eto-CM. Importantly, depletion of the Hsp70-HMGB1 complex from CM caused the down-regulation of the marker, while, of note, the addition of each pure protein alone did not increase the autophagy marker level and thus did not compensate for the absence of the complex (Fig. 5F).

### The Hsp70-HMGB1 complex is being formed inside the cells and then released to to extracellular milieu

**Fig. 6:**
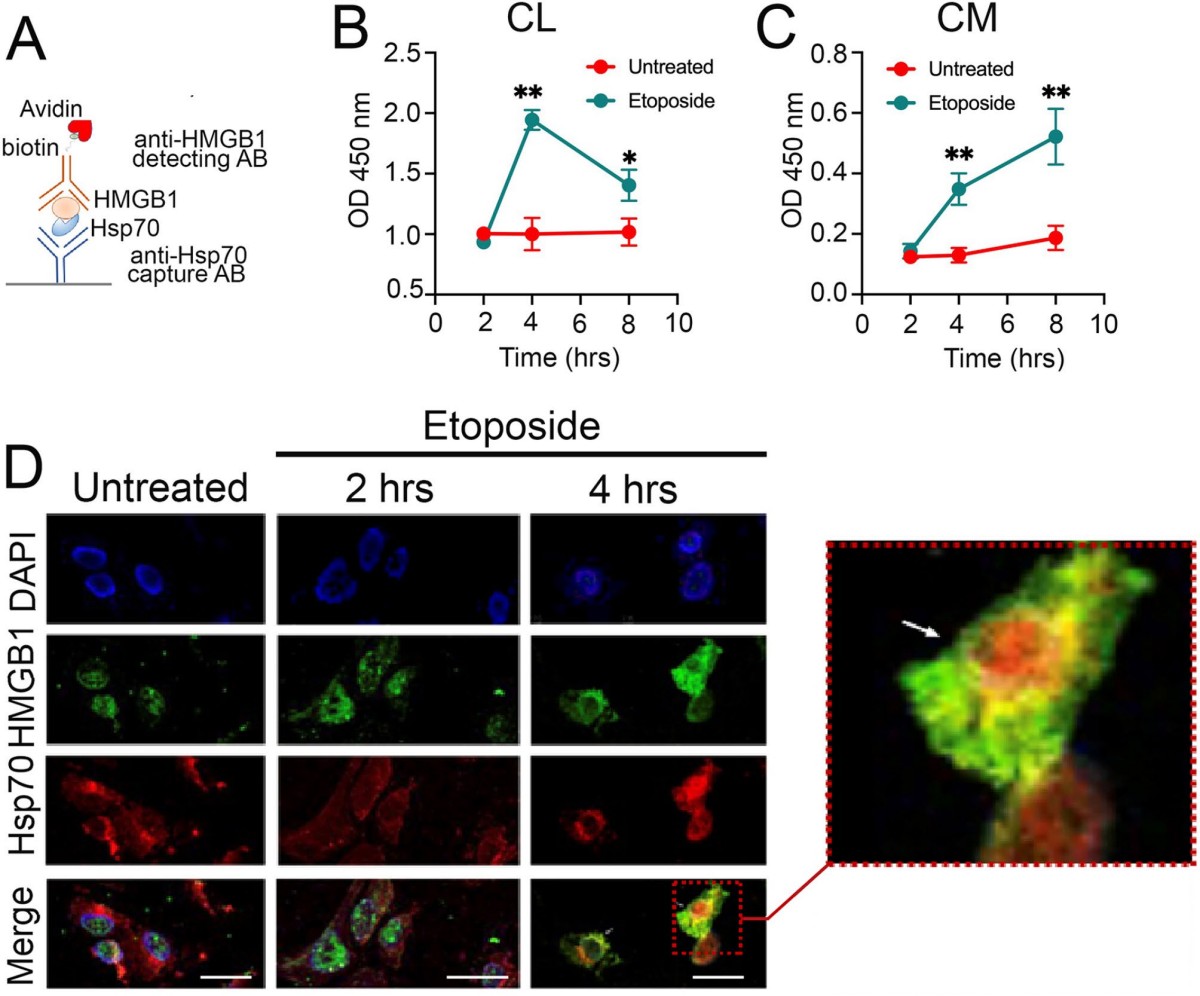
The Hsp70-HMGB1 complex is form inside cells and then releases to extracellular milieu. (**A**) Scheme of Protein-Protein Interaction assay (PPI). (**B,C**) A549 cell were treated with etoposide for 2, 4 and 8 h and then cell lysates (CL) (**B**) and cell media (CM) (**C**) were collected and amount of Hsp70-HMGB1 complexes was evaluated with PPI assay (as in scheme). (**D**) Data of confocal microscopy data for A549 cells treated with etoposide for 2 and 4 h and stained with antibodies against HMGB1 (green) and against Hsp70 (red). Nuclei were stained with DAPI. Scale bars: 10 μm

Next, we sought to study the features and dynamics of the complex formation using the PPI assay to quantify Hsp70-HMGB1 interactions in cells (CL) or in CMs taken from untreated A549 cells and treated with etoposide at various time points after treatment. Accordingly, to PPI assay data (see scheme on Fig. 6A), the maximum number of complexes in CL was found at 4 h after treatment, when its amount slightly reduced to time point of 8 h (Fig. 6B); in CM, the complex was detected at 4 h after treatment and at 8 h its amount increased by 3-fold compared to CM of untreated cells (Fig. 6C).

To further confirm the PPI data, we performed an experiment using confocal microscopy. A549 cells were seeded onto coverslips and treated with 100 µM of etoposide for 2 and 4 h. In untreated cells, HMGB1 was located in the nucleus, while Hsp70 was found in the cytoplasm. Interestingly, two hrs after treatment, HMGB1 migrated from nucleus to cytoplasm and 4 h later was found to be co-localized with Hsp70 (Fig. 6D, see also insert).

Taken together, these data prove that the Hsp70-HMGB1 complex is initially formed in tumor cells subjected to chemotherapy, before being released from the dying cells; in the extracellular milieu, the complex can stimulate the regrowth of tiny populations of tumor cells while each protein alone does not.

### Hsp70 inhibitors split Hsp70-HMGB1 complex and suppress tumor growth in vitro and in vivo

**Fig. 7:**
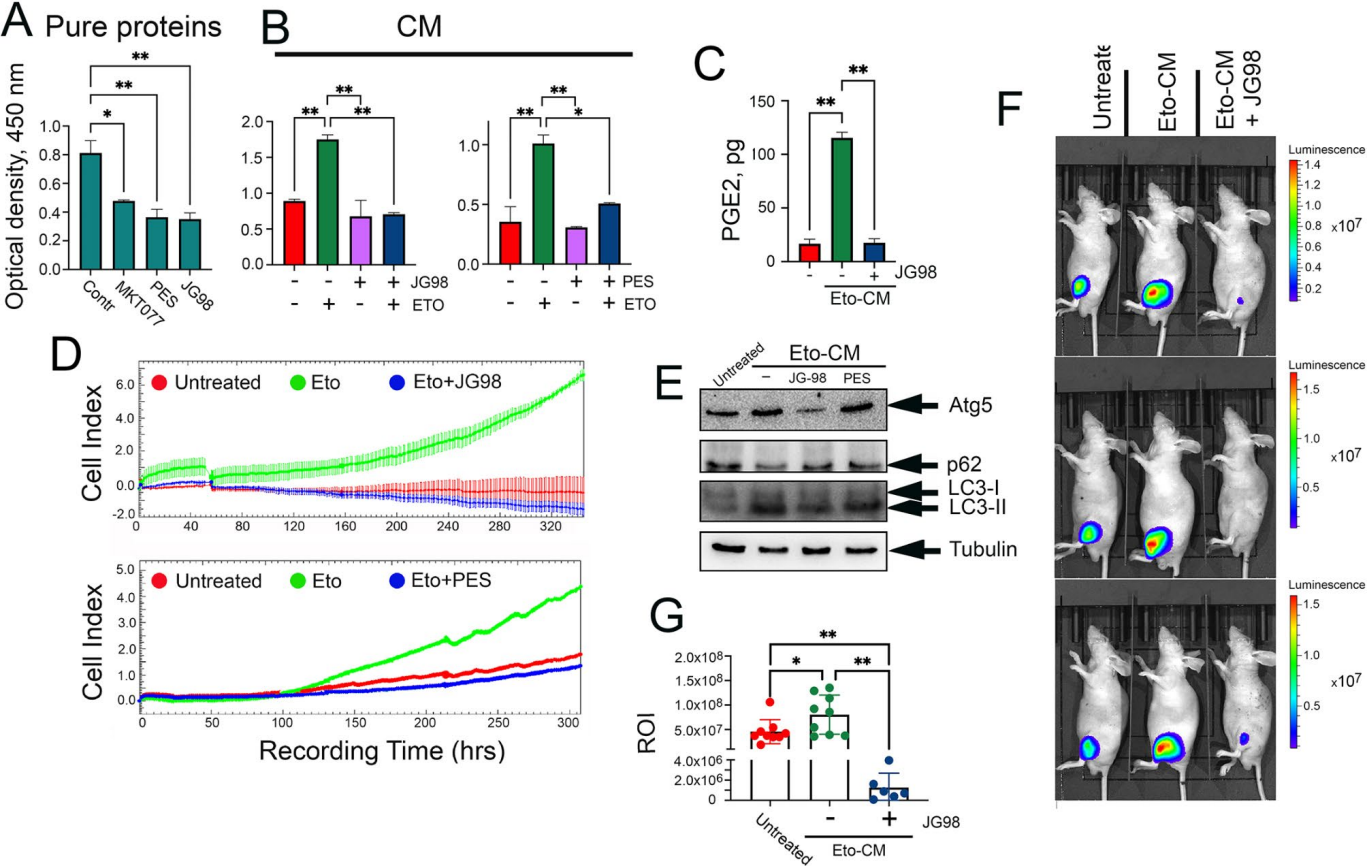
Hsp70 inhibitors reduce Hsp70-HMGB1 complexes formation in CM and suppress tumor cells growth in vitro and *in vivo.*(**A**) PPI assay of purified Hsp70 and HMGB1 in presence of known Hsp70 inhibitors. (**B**) The number of Hsp70-HMGB1 complexes in A549 cell media after treatment with etoposide in presence of JG-98 or PES. * p < 0.05; ** p < 0.001. (**C**) Proliferation of A549 cells incubated in CM of A549 cells treated with etoposide alone or in presence of JG98 (upper panel) or PES (lower panel) estimated with the use of xCELLigence in real time. (D) PGE2 production by A549 cells treated with Eto-CM and Eto + JG98-CM for 24 h (**E**) Autophagy level in A549 cells treated with CM from untreated cells, Eto-CM, Eto + JG98-CM and Eto + PES-CM. (**F**) A549 cells, untreated and incubated in Eto-CM or Eto + JG98-CM, were subcutaneously injected into Balb/c nude mice (n = 10 in each group) and 41 days later were monitored using the IVIS Spectrum imaging system. Representative bioluminescence images. (**G**) Luminescence of tumor lesions. * p < 0.05, ** p < 0.005

To test whether the uncoupling of Hsp70 from its complex with HMGB1 could affect repopulation, we utilized three small molecules inhibiting the Hsp70 chaperone activity that have been reported to exert antitumor effects: MKT-077, PES [32] and JG98 [33]. First, using the PPI assay with purified Hsp70 and HMGB1, we found that all three compounds were able to reduce the Hsp70-HMGB1 complex formation in a dose-dependent manner (Fig. 7A, Suppl Fig. 4). The maximal dissociating efficacy was demonstrated by PES and JG-98 (Fig. 7A), so they were further used for the treatment of A549 cells in combination with etoposide. We collected CMs 4 h after the treatment with the above combinations and using PPI assay, we found that both PES and JG-98 reduced the complex stability by 2-fold compared to full Eto-CM (Fig. 7B).

Next, we examined the repopulation-reducing activity of the aforementioned uncouplers by introducing them to the conditioned media; the following probes were used in this experiment: Eto-CM alone, Eto + PES-CM, Eto + JG-98-CM and CM from untreated A549 cells. Analysis of cell index data (xCELLigence) demonstrated that PES and to a greater extent JG-98, reduced the proliferation of A549 cells cultured in Eto-CM, suggesting that the dissociation of the Hsp70-HMGB1 complex was able to prevent repopulation (Fig. 7C). Using the MTT assay, we further proved that both uncouplers were able to inhibit the regrowth of sparse cell population. Moreover, the use of Oxa-CM alone or in the presence of PES or JG-98 also significantly reduced the repopulation of tumor cells (Suppl Fig. 5).

Next, we checked whether the mechanism by which the uncouplers impede tumor cell re-proliferation could be, at least partially, function via the reduction of PGE2 production and pro-survival autophagy marker, previously found to indicate the progress of repopulation. It was shown that JG-98 substantially inhibited PGE2 production by almost 10-fold (Fig. 7D) and, moreover, the compound significantly downregulated the pro-survival autophagy since the expression of ATG5 and LC3I-II decreased by 2- to 3-fold while p62 levels increased (Fig. 7E), all indicating the inhibitory effect of JG-98 on these repopulation-promoting pathways. Likewise, PES also decreased the expression of autophagy markers, but to a lesser extent than JG-98 did.

Lastly, since the use of PES instead of JG-98 resulted in a lower inhibitory-effect on Eto-CM-mediated repopulation (Fig. 7B, C, E), the compound was chosen for the experiments with animal models that were performed as described earlier (see Fig. 1D, E). We incubated luciferase-expressing A549 cells in following CMs: (i) from untreated A549wt cells, (ii) from cells treated with etoposide alone and (iii) from cells treated with a combination of etoposide and JG-98 for 7 days prior to injection into Balb/c nude mice. After 41 days, tumor size was evaluated with the aid of the IVIS Spectrum monitor. As expected, tumors obtained from A549_luc_ cells incubated with Eto-CM prior to injection were larger than tumors from untreated A549-luc cells. Consistently, tumors of A549-luc cells incubated in Eto-CM + JG-98 were significantly smaller (Fig. 7F, G), indicating that JG-98 also prevented the stimulatory effect of Hsp70-HMGB1 complex on tumor regrowth in vivo.

## Discussion

Cancer recurrence is an intensively explored field in modern biology that demonstrates the dynamic complexity of tumor organization and its regulation by a variety of molecular pathways related to tumor cells, tumor microenvironment (TME) and the immune system [23]. Furthermore, several reports have comprehensively demonstrated that, in addition to other extrinsic factors, dying cancer cells themselves are able to impact tumor relapse [12, 17, 34]. In our research, we first confirmed the results of these groups and secondly found that a factor that could stimulate the proliferation of a tiny population of tumor cells imitating so called minimal residual disease [19] can occur in the CM of cancer cells subjected to chemotherapy; this was established in both in vitro and in vivo experiments and formed the basis of our research model. Of note, autocrine regrowth effect, e.g. formed in the absence of TME or immune cell reaction, and based on the alternating mechanism of reversible poly- and de-polyploidization mechanism to survive radiotherapy was reported by Zhao et al. [35]. It is also important that a few of specific regulatory chains may modulate relationships between separate tumor cell sub-groups, and anaplastic lymphoma kinase is a protein able to make colon cancer cells to form populations with the enhanced recurrence potential [36].

Many tumors contain enhanced levels of Hsp70 that correlate with poor prognosis [37]. The protein can also be released into the extracellular milieu from living cells using an active transport mechanism [38], or it can leak from dying cells; therefore, in some instances, the protein concentration may become sufficient to affect physiology of neighboring tumor cells. It is clear that, once in the extracellular environment, the protein may function as a typical DAMP and in the context of this study to activate the proliferation of tumor cells. We tested this hypothesis using A549 and DLD1 wild-type control cells and ones devoid of the major part of the protein using shRNA-mediated knockdown and subjected them to anticancer drugs. The full Eto-CM and Oxa-CM with high Hsp70 levels significantly increased the growth of sparse populations of A549 and DLD1 cells when compared with cells incubated in CM from untreated cells or Eto-CM and Oxa-CM from cells with reduced Hsp70, which resulted in reduced cell proliferation. Data of colony formation assay and expression of cell proliferation markers, Aurka, Ki67 and MCM10, also demonstrated the decreased regrowth capacity of cells subjected to CM with a lower amount of Hsp70. We also found that the production of prostaglandin E, indicating regrowth activity [39], was dramatically reduced when we used CM taken from A549 cells with Hsp70 knockdown. To confirm the above data and investigate whether the protein alone can manage tumor cell repopulation, we employed two methods to remove Hsp70 from active CM, using an ATP-agarose gel specifically removing pure chaperone and immunoprecipitation with the application of specific antibodies.

Surprisingly, the application of ATP-agarose gel that allowed us to remove Hsp70 alone from Eto-CM and Oxa-CM did not affect the regrowth-stimulating activity of the latter, while immune-depletion of Hsp70 in both CMs led to a substantial reduction in repopulation. This finding confirmed our assumption about the important role of Hsp70 in repopulation but, at the same time, raised questions about the mechanism associated with the action of Hsp70.

Generally, Hsp70 release from cancer cells is considered a signal for dendritic cell maturation and the generation of antitumor-specific immune response [40, 41]. It is also well documented that exogenous Hsp70, like other DAMPs, is capable of inducing non-specific immunogenic cell death [42]. However, in our experiments, there were no components other than cancer cells of the same origin; therefore, one could consider the effect of Hsp70-enriched or depleted CM as evidence of the autocrine character of tumor cell repopulation. Since the immune response is mostly directed to eradicate large populations of fast-cycling cancer cells and because dormant or remaining after therapy-cells are mostly immunotolerant [43], the Hsp70-enhanced proliferation could be of the first steps in the tumor reawakening program.

The results of immunoaffinity depletion of Hsp70 show that the protein persists in CM in complex with other molecules; some of them interacted with the chaperone inside the cell and then emerged together in the extracellular space. The list of Hsp70 interactors has been presented in two large reports [44, 45].

Some of the proteins implied in the above interactomes are vital for tumor cell survival in its response to stressful or therapeutic regimes; some of which are focal adhesion kinase FAK [46], caspase 3/7 [47], Bim, Bcl-2 athanogene [48] and many others. Among the candidates for the role of Hsp70 binders, we also considered the high mobility group B1 protein, HMGB1 and calreticulin, both known DAMPs and markers of tumor relapse after radiotherapy [49]. To identify the proteins binding to Hsp70 and whose activity could be modulated by this interaction, we employed immunoprecipitation on Eto-CM of A549 cells. Using a proteomic approach, we identified HMGB1 as an Hsp70-binding protein. Recently, extracellular HMGB1 was established to bind with RAGE, stimulate the development of drug resistance after chemotherapy and regulate autophagy and apoptosis in neighboring tumor cells [50, 51]; the protein has also been proposed as a non-invasive marker for the detection of bladder carcinoma. Very recently HMGB1 released from dead tumor cells was proved to be a trigger of repopulation of the residual hepatocellular carcinoma cells after radiotherapy [52].

In our study, we proved (for the first time) the presence of the Hsp70-HMGB1 complex using FRET assay that demonstrated a dose-dependent increasing signal, in addition to Protein-Protein Interaction assay, immunoprecipitation and pull-down assays. Interestingly, irrespective of how we pulled out the complex from Eto-CM, via fishing Hsp70 with specific antibodies or HMGB1 using HBHP-binding peptide, the result was the same: a reduction in A549 tumor cell repopulation. Of note, the addition of pure recombinant Hsp70 or HMGB1 to their complex-depleted Eto-CM did not affect the suppression of repopulation. However, when we added the complex to the depleted conditioned media, repopulation was restored. Several previous studies have been shown that exogenous HMGB1 promotes pro-survival autophagy and stimulates the tumor growth of gliomas [53] and esophageal squamous carcinoma [29], in addition to enhancing the chemoresistance of tumor cells in leukemic and bladder cancers [50]. Hence, we checked the expression of autophagy markers in A549 cells treated with either full Eto-CM or with Eto-CM depleted by specific anti-Hsp70 antibodies or with HMGB1-binding HBHP peptide. Accordingly, we found that any kind of depletion caused a reduction inLC3 I-II expression levels compared with full Eto-CM and the addition of either Hsp70 or HMGB1 to depleted media did not increase LC3 I-II expression. All of these data convinced us that the Hsp70-HMGB1 complex can stimulate compensatory growth in tumor cell populations. It was previously reported that HMGB1 is able to promote tumor progression in prostate cancer [54] and pancreatic cancer [55] and enhance chemoresistance in acute leukemia [50] cells. Here we demonstrate that HMGB1 needs to be in complex with Hsp70 to perform the aforementioned pro-tumorigenic functions.

We also demonstrated that the Hsp70-HMGB1 complex was formed in the cytoplasm when, due to antitumor drug, HMGB1 exported from nucleus and encounters in cytoplasm Hsp70, the amount of which was also increased in response to therapy. The release of the protein into the cytoplasm can be due to its phosphorylation as demonstrated in RAW264.7 mouse macrophages and human monocytes [56]. Earlier Bonardi et al. showed that HMGB1 acetylation may induce its relocation to cytosol and again in mouse and human macrophages [57]. More recently, N37, N134 and N135 residues of HMGB1 were found to be N-glycosylated, and this modification strongly affected DNA-binding ability of the protein and its nucleo-cytoplasmic transport [61].

Finally, we performed a search for uncouplers of the Hsp70-HMGB1 complex among known Hsp70 chaperone inhibitors, MKT077, PES [32] and JG-98 [58]. As a result, in the PPI assay, PES and JG98 significantly reduced the number of Hsp70-HMGB1 complexes in experiments with pure proteins and also in Eto-CM; however, in the xCelligence test, Eto-CM collected from cells additionally treated with JG98 substantially decreased the cell index and also PGE2 production was remarkably lower. The use of Eto-CM collected from cells after JG-98 treatment also suppressed autophagy, which was evaluated by an increase in the p62 expression and a decrease in ATG5 and LC3 I-II. Moreover, when mice were injected with A549 cells incubated in Eto + JG-98-CM, tumors grown in nude mice had much smaller sizes than those taken from the cells incubated with the conditioned medium of cells treated with etoposide only. In conclusion, our results presented here indicate that the regrowth of tiny populations of tumor cells may be triggered by a complex of two DAMPs, Hsp70 and HMGB1, marking a wide variety of relapsing tumors. In addition, the Hsp70-HMGB1 complex could be of great relevance as a marker of tumor recurrence; moreover, the complex might serve as a promising therapeutic target for suppressing tumor relapse, as demonstrated by JG-98.

## Conclusion

Activation of compensatory cell proliferation often observed after surgery or radio-, chemotherapy occurs due to functions of a few of so-called alarmins. In this work we demonstrated that two proteins with the pronounced pro-tumor activity Hsp70 and HMGB1 form complex in cells, subjected to chemotherapy and being released to extracellular space trigger proliferation of a tiny population of cancer cells. The compounds able to split the complex inhibit tumor regrowth in cell and animal models of tumor relapse proving that the interaction between the two alarmins can be a marker of a tumor relapse and administration of Hsp70 inhibitors may become an additional tool to avoid a risk of tumor relapse.

### Electronic supplementary material

Below is the link to the electronic supplementary material.

Supplementary Material 1

## Data Availability

The datasets used and analysed during the current study are available within the manuscript and its additional files.

## Abbreviations

Aurka: Aurora kinase A
ATP: Adenosine triphosphate
CM: Conditioned medium
CSCs: Cancer stem cells
DAMPs: Damage-associated molecular patterns
ELISA: Enzime-linked immunosorbent assay
Eto: CM-conditioned medium from etoposide treated cells
FRET: Förster resonance energy transfer
HBHP: HMGB1 binding heptamer peptide (HMSKPVQ)
HMGB1: The High Mobility Group B1
Hsp70: heat shock protein 70
IP: Immunoprecipitation
MCM: 10-mini-chromosome maintenance protein 1
Oxa: CM-conditioned medium from oxaliplatin treated cells
PGE: Prostaglandin E
PPI: Protein-Protein Interaction assay
qPCR: Quantitative
TME: Tumor microenvironment
