# Supplementary material for: Autocrine regulation of tumor cell repopulation by Hsp70-HMGB1 alarmin complex
Source: J Exp Clin Cancer Res. 2023 Oct 25;42:279. doi: 10.1186/s13046-023-02857-0 (PMC10598926; doi:10.1186/s13046-023-02857-0)
Supplement: Supplementary file 1 — Supplementary Material 1 [file 13046_2023_2857_MOESM1_ESM.pdf]

Supplementary Material
to the manuscript “Supplementary Material
to the manuscript “Autocrine regulation of tumor cell repopulation by Hsp70-HMGB1 alarmin complex”
by Sverchinsky et al

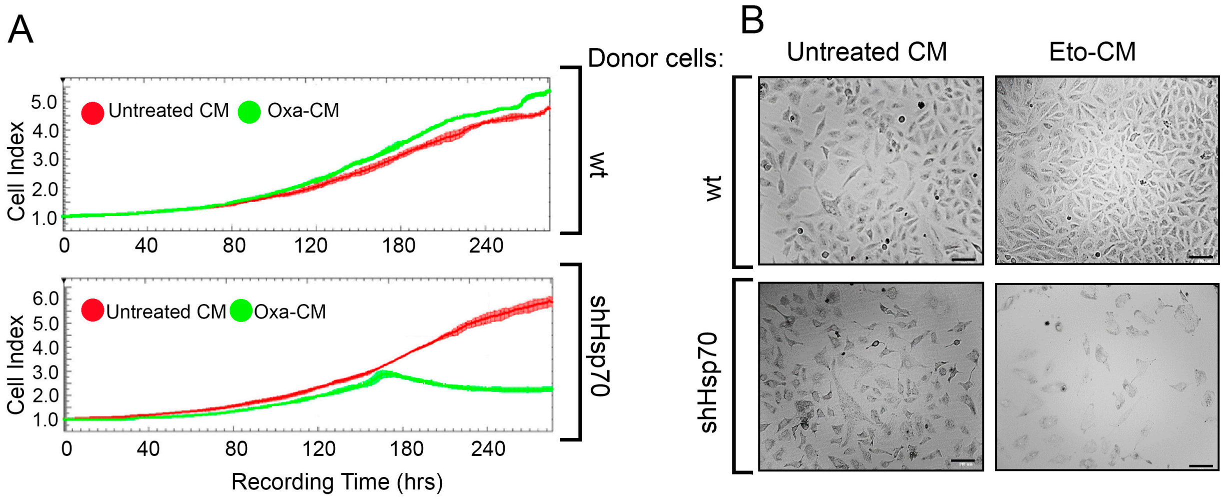

Figure 1S. **Hsp70 occurring in CM enhances the repopulation of H1299 human lung carcinoma cells.**
(A) Data of xCELLigence experiment on H1299 cell growth in Oxa-CM from H1299wt and H1299shHsp70 cells. (**B**) The microscopy of H1299 cells growing in Eto-CM from H1299wt and H1299shHsp70 cells for 14 days. Scale bar - 50 µm.

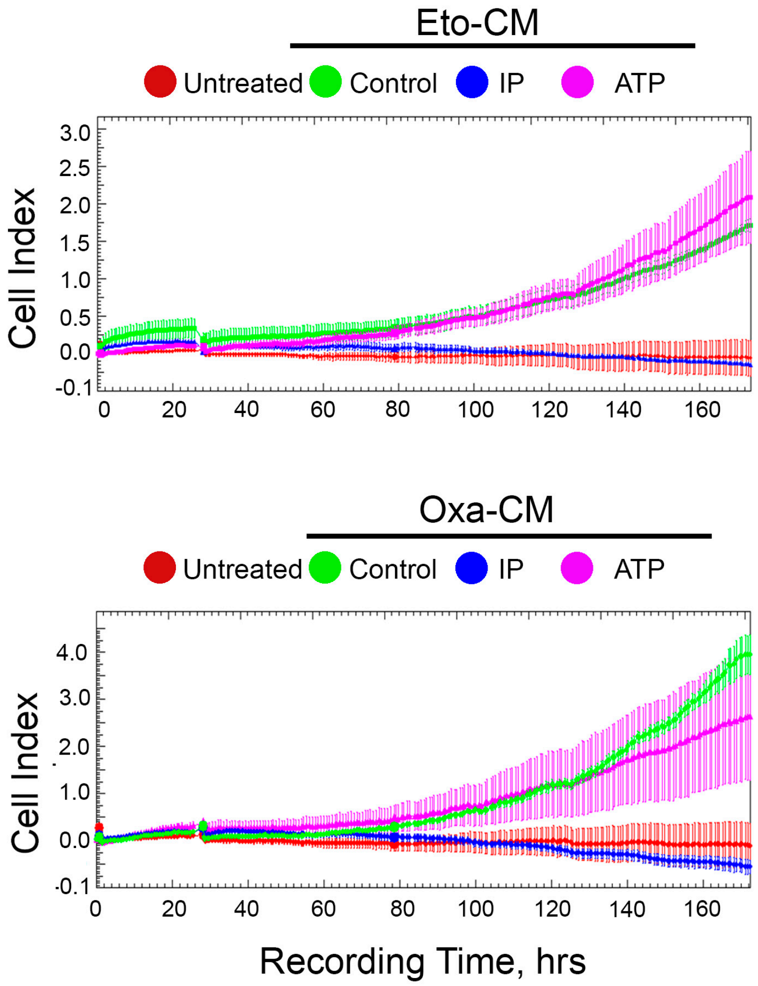

Figure 2S. **Depletion of Hsp70 from Eto-CM or Oxa-CM with the aid of ATP or IP with anti-Hsp70 results in distinct effect on DLD1 cell repopulation rate.**: ATP depletion did not affect the increase of cell growth caused by Eto-CM or Oxa-CM whereas the depletion with specific antibody canceled growth-stimulating properties of CM.

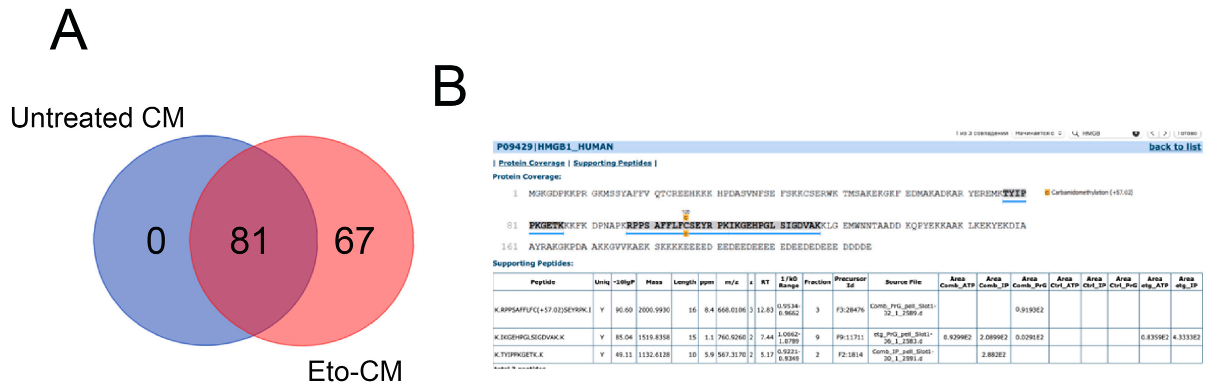

Figure 3S. Proteomic analysis revealed High mobility box group B1(HMGB1) among 67 proteins bound to Hsp70 released from Eto-treated A549 cells but not to Hsp70 released from untreated cells.

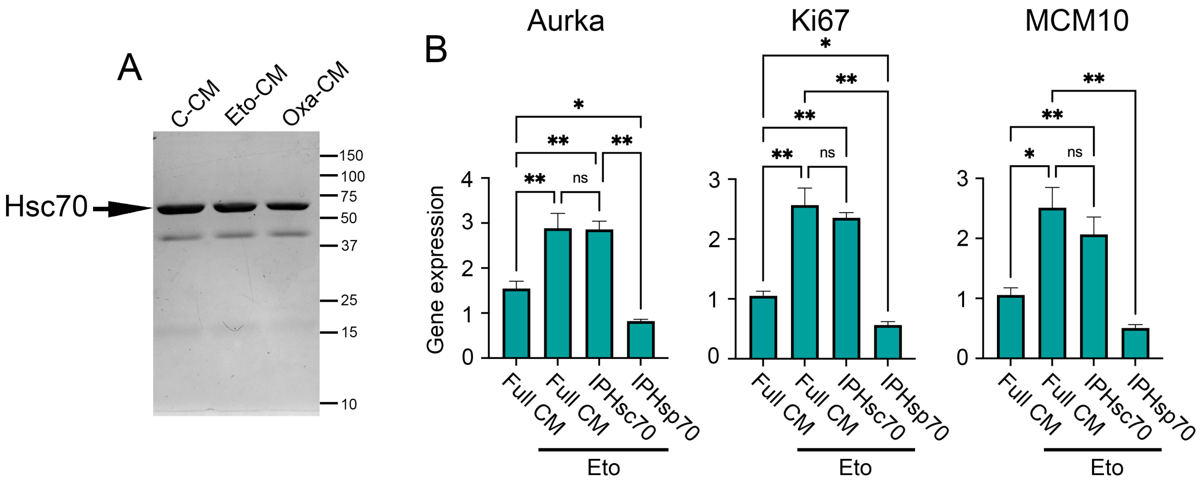

Figure 4S. **Removal of Hsc70 from the conditioned medium of A549 cells treated with etoposide does not lead to the abolition of repopulation.**

(A) Ag-stained gel from CM from untreated A549 cells and from Eto-CM and Oxa-CM performed with antibodies against Hsc70. (B) Expression of repopulation marker genes in A549 cells in presence of Eto-CM depleted with Hsc70. *p < 0.01; **p < 0.001.

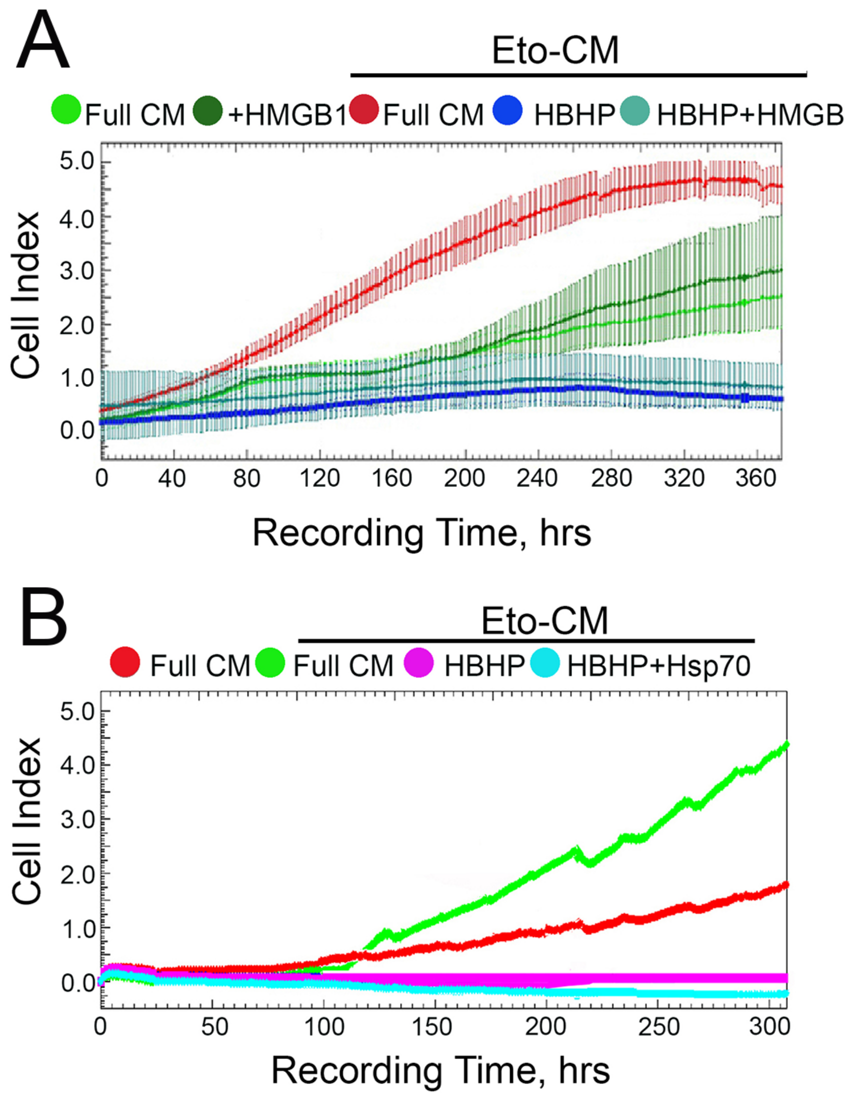

Figure 5S. **Hsp70 or HMGB1 separately do not stimulate tumor cell repopulation**

HMGB1-Hsp70 complex formed in Eto-CM from A549 cells was removed by the addition of HBHP-Biotin-Avidin gel, then purified HMGB1 (A) or purified Hsp70 (B) were added and these media were applied to A549 seeded to E-plates at a low concentration. Cell growth in real time was estimated using the xCELLigence technique.

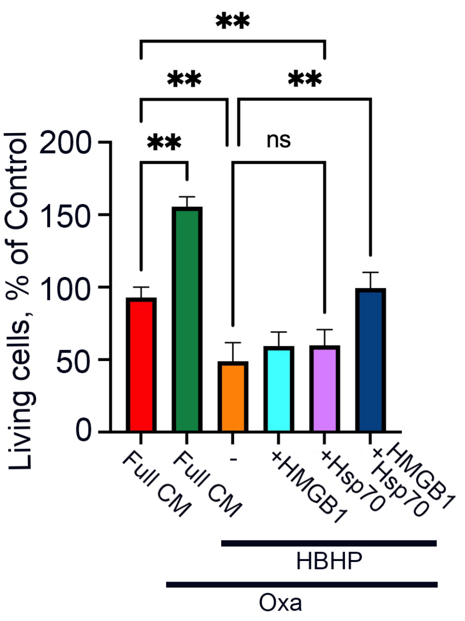

Fig. 6S. **To stimulate tumor cell repopulation, Hsp70-HMGB1 complex is needed.**
HMGB1-Hsp70 complex was depleted from Eto-CM from A549 cells with aid of HBHP-Bio-Avidin gel and then purified HMGB1 or purified Hsp70 or Hsp70-HMGB1 complex were added and these media were applied to A549 seeded to plates at a low concentration. Twelve days later, cell growth was assessed using the MTT method. **p < 0.001.

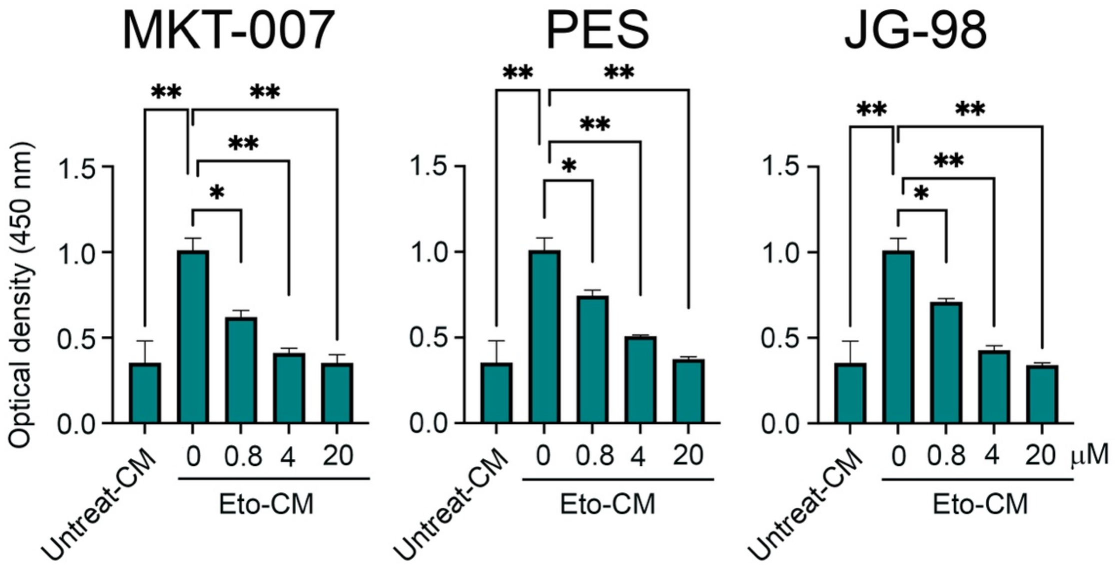

Figure 7S. **Uncouplers of Hsp70-HMGB1 complex reduced the Hsp70-HMGB1 complex formation dose-dependently**. Data of PPI assay with purified Hsp70 and HMGB1in the same mode as on Fig. 4E.

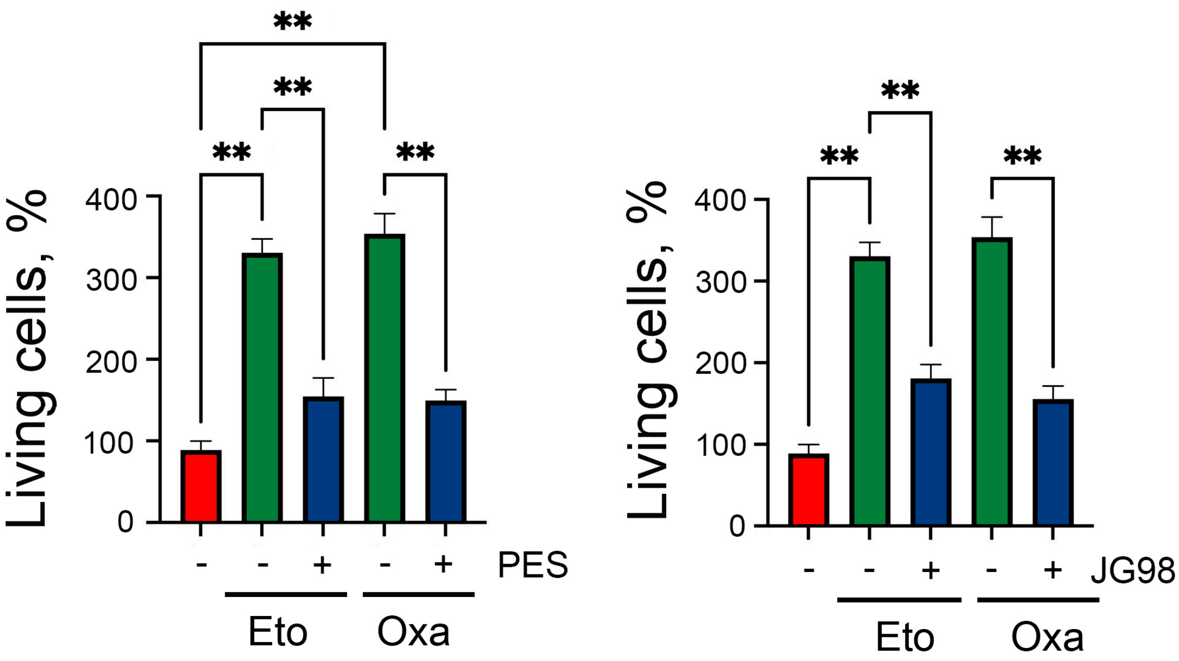

Figure 8S. **Hsp70 inhibitors able to reduce Hsp70-HMGB1 complexes formation in CM suppress tumor cells growth *in vitro***

A549 cells were seeded into wells of 96-well plates at a low concentration and incubated in Eto-CM or in Oxa-CM in presence of PES (left) or JG-98 (right) during 14 days. Data of MTT assay. ** $p < 0.001$.
